# Kinetics, Isotherm and Thermodynamic Studies for Efficient Adsorption of Congo Red Dye from Aqueous Solution onto Novel Cyanoguanidine-Modified Chitosan Adsorbent

**DOI:** 10.3390/polym13244446

**Published:** 2021-12-18

**Authors:** Nouf F. Al-Harby, Ebtehal F. Albahly, Nadia A. Mohamed

**Affiliations:** 1 Department of Chemistry, College of Science, Qassim University, P.O. Box 6644, Buraydah 51452, Saudi Arabia; e.albahly@qu.edu.sa (E.F.A.); NA.AHMED@qu.edu.sa (N.A.M.); 2 Department of Chemistry, Faculty of Science, Cairo University, Giza 12613, Egypt

**Keywords:** cyanoguanidine-modified chitosan adsorbent, Congo red dye, adsorption kinetics, adsorption isotherms, adsorption thermodynamic

## Abstract

Novel Cyanoguanidine-modified chitosan (CCs) adsorbent was successfully prepared via a four-step procedure; first by protection of the amino groups of chitosan, second by insertion of epoxide rings, third by opening the latter with cyanoguanidine, and fourth by restoring the amino groups through elimination of the protection. Its structure and morphology were checked using Fourier-transform infrared spectroscopy (FTIR), X-ray diffraction (XRD) and scanning electron microscopy (SEM) techniques. The adsorption capacity of CCs for Congo Red (CR) dye was studied under various conditions. It decreased significantly with the increase in the solution pH value and dye concentration, while it increased with increasing temperature. The adsorption fitted to the pseudo-second order kinetic model and Elovich model. The intraparticle diffusion model showed that the adsorption involved a multi-step process. The isotherm of CR dye adsorption by CCs conforms to the Langmuir isotherm model, indicating the monolayer nature of adsorption. The maximum monolayer coverage capacity, q_max_, was 666.67 mg g^−1^. Studying the thermodynamic showed that the adsorption was endothermic as illustrated from the positive value of enthalpy (34.49 kJ mol^−1^). According to the values of ΔG°, the adsorption process was spontaneous at all selected temperatures. The value of ΔS° showed an increase in randomness for the adsorption process. The value of activation energy was 2.47 kJ mol^−1^. The desorption percentage reached to 58% after 5 cycles. This proved that CCs is an efficient and a promising adsorbent for the removal of CR dye from its aqueous solution.

## 1. Introduction

In recent years, many pollutants, especially synthetic dyes, have flowed into the water environment. A small quantity of these dyes in water is very clear and dangerous to aquatic life. In addition, dyes can lead to severe damage to the liver, digestive and central nervous systems of humans. [1,2,3]. Due to the synthetic origin and the complex structure of these dyes, they are stable in light and heat and are not bio-degradable [4]. Many techniques have been applied to remove dyes, including biodegradation, membrane filtration, coagulation-aggregation, electrochemical and oxidation processes. These techniques have a considerable efficiency, but they face some issues, including high energy consumption, a large cost, a large amount of toxic residues and poor performance at low dye concentrations [1,2,3,4].

Adsorption methods have been proven to be a highly effective technique for the removal of dyes due to their simplicity, easy operation, low initial cost, and the adsorbent abundance with no undesirable secondary product, in addition to easy restoring of the adsorbents for reuse [1,2,3,4,5]. The most important adsorbent is the carbon because of its high efficiency to remove the various contaminants, but it is highly expensive and is difficult in regeneration [6]. Therefore, it is necessary to find other effective and low-cost adsorbents. The use of adsorbents based on natural biopolymers has attracted considerable attention, especially chitosan and its derivatives.

Chitosan, a natural linear polysaccharide, is a non-toxic, eco-friendly, antimicrobial, biocompatible and biodegradable material [7,8,9]. Chitosan is one of the most promising adsorbents due to its low initial cost, ease of operation and efficient removal of dyes compared with activated carbon and other adsorbents. Because of its unique polycationic nature, it has a high capacity to remove anionic dyes through protonation processes of its amino groups in acidic medium. However, the use of chitosan as an adsorbent is limited due to its low surface area, the formation of colloid in water and its deterioration by chemical and microbial procedures. In addition, the high dissolution ability of chitosan in acidic media is considered to be its greatest shortcoming, especially in the case of its usage as an adsorbent for the removal of dyes since the effluent is usually acidic. Thus, modification of chitosan via grafting [2,10,11,12,13], blending [14,15,16] and cross linking [17,18,19,20,21,22] is used to improve chitosan properties, such as an increase of its functionality, and a lowering of its solubility, inhibiting its degradation rate and increasing the life span of its products in different media.

In this study, to avoid the aforementioned drawbacks of chitosan and to improve its adsorption capacity for anionic dyes, chitosan was chemically modified via prior protection of its primary amino groups using benzaldehyde. Thus, the reaction of epichlorohydrin was confined to the primary hydroxyl groups on C6 of the produced chitosan Schiff’s base derivative, to incorporate epoxy moieties which can be readily opened by compounds possessing a free electron pair as cyanoguanidine. Afterwards, benzaldehyde was eliminated in an acidic medium and the primary amino groups on chitosan were regained to obtain cyanoguanidine-modified chitosan. It would be expected that the inclusion of nitrogen-rich cyanoguanidine moieties into the repeating units of chitosan, in addition to the regained amino groups of chitosan, will increase the basic sites available for adsorption the anionic dyes as CR dye. The adsorption kinetic was studied under the influence of different variables, such as initial dye concentration, temperature, time period and pH of the adsorption medium to reach the ideal conditions of adsorption and to determine the optimum adsorption capacity. The thermodynamic parameters, such as enthalpy, entropy and the free energy of adsorption, were determined. The possibility of regeneration of the modified biomaterial for reuse was also studied.

## 2. Materials and Methods

### 2.1. Materials

Chitosan (1.0–3.0 × 10^5^ g mol^−1^ and 98% deacetylation degree) was obtained from Acros Organics (Newark, NJ, USA). Benzaldehyde and epichlorohydrin were purchased from PanReac. AppliChem- ITW Reagent (Darmstadt, Germany). Cyanoguanidine was supplied by Sigma-Aldrich (Munich, Germany). CR dye was supplied by Winlab (Leicestershire, UK). The other chemicals and solvents were obtained from Sigma-Aldrich (Munich, Germany).

### 2.2. Preparation of Novel Cyanoguanidine-Modified Chitosan (CCs) Adsorbent

First step: chitosan Schiff’s base should be prepared via reaction of chitosan with benzaldehyde to direct the chemical modification to the primary -OH groups on chitosan. The suspended chitosan (5 g) that swollen in 50 mL methanol was well stirred with 20 mL of benzaldehyde at room temperature for 24 h. The produced chitosan Schiff’s base has been filtered, rinsed repeatedly with MeOH and dried at 50 °C to constant weight [21].

Second step: 4 g of chitosan Schiff’s base were suspended and well stirred in aqueous sodium hydroxide solution (120 mL, 0.001 mol L^−1^) at room temperature for 15 min for swelling. To the suspended solution, 10 mL of epichlorohydrin were slowly added with continuous stirring for a further 6 h. The yielded epoxy chitosan Schiff’s base has been filtered, rinsed repeatedly with H_2_O and dried at 50 °C to constant weight [22].

Third step: 2 g of cyanoguanidine were dissolved in 25 mL of water, then were slowly mixed with a solution of epoxy chitosan Schiff’s base (2 g) that swelled and was suspended in 60 mL of aqueous sodium hydroxide solution (0.001 mol L^−1^) and stirred at room temperature overnight. The produced cyanoguanidine chitosan Schiff’s base was obtained by filtration, washing frequently with methanol as well as acetone and drying at 50 °C to constant weight.

**Scheme 1:**
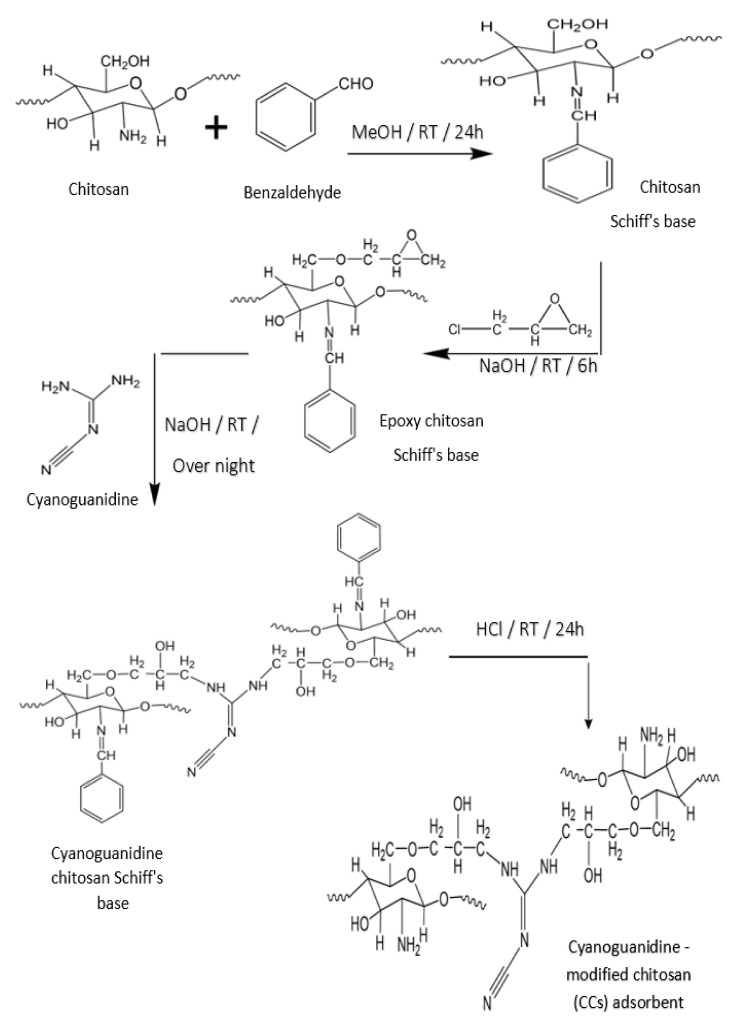
Preparation of novel CCs adsorbent.

Forth step: to remove the protection from the NH_2_ groups, cyanoguanidine chitosan Schiff’s base (2 g) was treated with 60 mL of hydrochloric acid in ethanol (0.24 mol L^−1^ HCl) and stirred at room temperature for 24 hrs. The formed cyanoguanidine-modified chitosan (CCs) adsorbent was neutralized with aqueous sodium carbonate solution (1 wt%) till pH 7, filtered, washed frequently with ethanol and dried at 50 °C to constant weight (Scheme 1).

### 2.3. Measurements

#### 2.3.1. FTIR Spectroscopy

A Thermo Scientific Nicolet 6700 FTIR spectrometer (Tokyo, Japan) was utilized to record FTIR spectra of the modified chitosan derivatives using KBr pellets in the wave number range of 4000–500 cm^−1^.

#### 2.3.2. X-ray Diffractometry

A Rigaku Ultima-IV wide-angle X-ray diffractometer (Tokyo, Japan) was used to study the morphology of the modified chitosan derivatives at diffraction angles (2θ) at a range between 5 and 80° with a speed of 5° min^−1^.

#### 2.3.3. Scanning Electron Microscopy

A field emission scanning electron microscope JSM-7610F (Freising, Germany) was used to photograph the surface topography of the modified chitosan derivatives after coating with a thin layer of gold at an accelerating voltage of 15 kV and at a magnification of 8000×.

### 2.4. Adsorption Studies

#### 2.4.1. Standard Curve of CR Dye

Anionic CR dye was chosen due to its extensive applications. Its stock solution was prepared by dissolution of CR dye in double distilled water of a concentration of 1000 mg L^−1^. The prepared stock solution was then diluted into 10 different dye concentrations using double distilled water. The absorbance of each dye solution was measured using UV–vis spectrophotometer (Shimadzu UV/Vis 1601 spectrophotometer, Kyoto, Japan) at λ_max_ = 497 nm. A calibration curve was plotted between concentration and absorbance using the predetermined concentrations of CR dye. The molar absorptivity was determined using Beer–Lambert law Equation (1).

**Figure 1:**
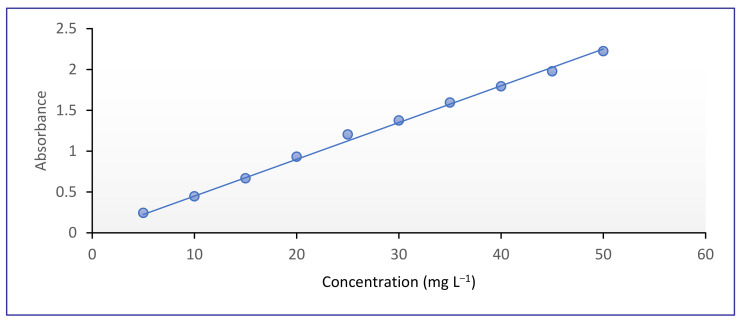
Absorption coefficient of CR dye.

A = εlc(1)

where A is the absorbance, ε is the molar absorptivity (L mol^−1^ cm^−1^), l is the path length of the cuvette that containing the sample (cm) and c is the concentration of dye in solution (mg L^−1^). The molar absorptivity of CR dye was 0.045 L mol^−1^ cm^−1^ (Figure 1).

#### 2.4.2. Adsorption of CR Dye Using CCs Adsorbent

The removal of CR dye by CCs adsorbent was studied using batch experiment. 50 mL of dye solution was added into an Erlenmeyer flask with 50 mg of the absorbent. The solution was then shaken in a water bath shaker (70 rpm) until the equilibrium was attained. The residual dye concentration was analyzed spectrophotometrically at λ_max_ = 497 nm.

The amount of adsorbed dye onto adsorbent at certain time, q_t_ (mg g^−1^), and at equilibrium, q_e_ (mg g^−1^), were calculated using Equations (2) and (3), respectively.

(2) $$q_{t}=\frac{\left(C_{o}-C_{t}\right)\;V}{W}$$

(3) $$q_{e}=\frac{\left(C_{o}-C_{e}\right)\;V}{W}$$

where q_t_ and q_e_ are the amount of adsorbed dye (mg g^−1^) at time t and at equilibrium, respectively, C_o_ is the initial concentration of the dye (mg L^−1^), C_t_ is the dye concentration at time t (mg L^−1^), V is the volume of the dye solution (L) and W is the weight of the adsorbent (g).

The removal efficiency (R.E.) of dye can be calculated by Equation (4).

(4) $$\%\;R.E.=\frac{\left(C_{o}-C_{e}\right)}{C_{o}}\times 100$$

where C_o_ is the initial concentration of the dye (mg L^−1^) and C_e_ is the equilibrium concentration of the dye (mg L^−1^).

Batch experiments were performed in stoppered Erlenmeyer flasks at various pH values (4, 7 and 9) using 50 mL of CR dye solution (600 mg L^−1^) and 50 mg of adsorbent. The solutions were continuously shaken at 70 rpm in a water bath shaker at 55 °C. For pH adjustment, HCl and NaOH solutions were utilized.

For studying the effect of temperature, 50 mg of CCs adsorbent was added to 50 mL of CR dye solution (600 mg L^−1^) at different temperatures (25, 35, 45 and 55 °C) at pH 9.

Studying the effect of CR dye concentration is carried out by adding 50 mg of CCs adsorbent to 50 mL of dye solution of various concentrations (400, 500, 600 and 1000 mg L^−1^) at pH 4 and 55 °C.

#### 2.4.3. Kinetic Studies

Studying the kinetics of the adsorption process is very significant for understanding the adsorption rate onto the particle surface, since adsorption kinetics show the influence of different conditions on the speed of the process by using models that could describe this reaction. In addition, adsorption kinetics determine the mechanism of dye adsorption onto the adsorbent material.

The kinetic data of CR dye were modeled using four different kinetic models: pseudo-first order, pseudo-second order, Elovich and intraparticle diffusion models.

##### Pseudo-First-Order Model (Lagergren Model)

This kinetic model determines the relationship between the change in time and the adsorption capacity with order of one. It is expressed by Equation (5), where q_t_ and q_e_ are adsorption capacity at time t and at equilibrium time, respectively, and k_1_ is the rate constant.

(5) $$\frac{{\mathrm{dq}}_{t}}{\mathrm{dt}}=k_{1}\left(q_{e}-q_{t}\right)$$

Integrating Equation (5) with boundary conditions of q_t_ = 0 at t = 0 and q_t_ = q_t_ at t = t gives a linear equation of pseudo-first order, Equation (6).

(6) $$\log\left(q_{e}-q_{t}\right)\;=\log q_{e}-\frac{k_{1}}{2.303}t$$

where q_e_ and q_t_ are the adsorption capacity at equilibrium time and time t (mg g^−1^), respectively, k_1_ is the pseudo first order rate constant (min^−1^) and t is the time (min). The values of q_e_ and k_1_ can be determined from the intercept and the slope of the linear plot of log (q_e_ − q_t_) versus t.

##### Pseudo-Second-Order Model (Ho and Mckay Model)

The pseudo-second-order kinetic model is expressed by Equation (7), which shows the relationship of the adsorption capacity and concentration with second order. This model describes the adsorption of the dissolved ions of the dye, via a cation exchange or chemical sharing, onto the adsorbent surface, assigning that a chemical process is involved during the adsorpt.

(7) $$\frac{{\mathrm{dq}}_{t}}{\mathrm{dt}}=k_{2}{\left(q_{e}-q_{t}\right)}^{2}$$

The integrated form of Equation (7) gives the linear form of pseudo-second order equation, Equation (8).

(8) $$\frac{t}{q_{t}}=\frac{1}{k_{2}q_{e}^{2}}+\frac{t}{q_{e}}$$

where q_e_ and q_t_ are the amount of adsorbed dye onto adsorbent at equilibrium time and time t (mg g^−1^), respectively, k_2_ is the pseudo-second order constant (g mg^−1^ min^−1^). The slope and intercept of the linear plot of t/q against t yielded the values of q_e_ and k_2_, respectively [23].

##### Elovich Model

This model is an interesting one for describing the activated chemisorption process, since it is generally applicable for chemisorption kinetics. It can cover a large range of slow adsorption processes. It is valid for the heterogenous adsorbent surfaces and expressed by Equation (9).

(9) $$\mathrm{qt}\;=\frac{1}{\beta }\ln(\alpha \beta )+\frac{1}{\beta }\;\ln\;t$$

where α is the initial adsorption rate constant (mg g ^−1^ min^−1^), β is the desorption constant related to the chemisorption activation energy and the surface coverage (g mg^−1^), and q_t_ is the adsorbed dye (mg g^−1^) at time t (min). By plotting q_t_ versus ln t, a straight line is obtained where values of α and β can be obtained.

##### Intraparticle Diffusion Model (Webber and Morris Model)

This model is used to determine the rate-controlling for the adsorption process and is expressed by Equation (10).

q_t_ = (k_int_t^1/2^) + C (10)

where k_int_ is the intraparticle diffusion constant (mg g^−1^ min^−1/2^), C is a constant (mg g^−1^) that is directly proportional to the boundary layer thickness. Both values of k_int_ and C can be obtained by calculations from slope and intercept resulted from the linear curve of q_t_ versus t^1/2^, respectively [24].

##### Kinetic Validation

To determine the most applicable model for describing the adsorption process, the normalized standard deviation Δq_e_ (%) is used and calculated using Equation (11) [25].

(11) $$\Delta q_{e}\;(\%)=100\;\times \;\sqrt{\frac{{\left[\left(q_{t,\exp}-{\;q}_{t,\mathrm{cal}}\right)/q_{t,\exp}\right]}^{2}}{N-1}}$$

where q_t,exp_ is the experimental adsorption capacity (mg g^−1^), q_t,cal_ is the calculated adsorption capacity for pseudo-first and pseudo-second models (mg g^−1^) and N is the number of data points.

#### 2.4.4. Adsorption Isotherm for CR Dye

Studying the adsorption isotherm is important for describing the ability of adsorbate molecules to distribute between the liquid and the solid phases at equilibrium state of the adsorption process. For studying adsorption isotherm, 10 mL of dye solution (400–1000 mg L^−1^) was shaken with 10 mg of adsorbent in a shaking water bath (70 rpm) at 55 °C and pH 9. After reaching the equilibrium, the concentrations of un-adsorbed dye were determined by measuring absorbance of dye solution using UV-vis spectrophotometer at λ_max_ = 497 nm. The adsorption isotherm was studied using four models; Langmuir, Freundlich, Temkin and Dubinin–Radushkevich models.

##### Langmuir Isotherm Model

Langmuir model supposes the formation of a monolayer coverage of adsorbate molecules onto a homogenous surface of adsorbent without interaction between the adsorbate molecules. This isotherm can be expressed by Equation (12).

(12) $$\frac{C_{e}}{q_{e}}=\frac{1}{\left(q_{\max}.K_{L}\right)}+\frac{C_{e}}{\left(q_{\max}\right)}$$

where q_e_ is the amount of adsorbed dye at equilibrium (mg g^−1^), C_e_ is the equilibrium concentration of dye in solution (mg L^−1^), q_max_ is the maximum monolayer coverage adsorption capacity (mg g^−1^), and K_L_ is a Langmuir coefficient that is concerned with adsorption energy (L mg^−1^). By plotting C_e_/q_e_ versus c_e_, results in a linear relationship, the values of both q_max_ and K_L_ can be obtained from the slope (1/q_max_) and the intercept (1/(q_max_ K_L_)), respectively. There is an important parameter for Langmuir model which is the separation factor (R_L_), Equation (13).

(13) $$R_{L}=\frac{1}{\left(1+K_{L}C_{o}\right)\;}$$

where K_L_ is the Langmuir constant and *C_o_* is the initial concentration of the adsorbate in solution. This parameter indicates whether the process is irreversible, favorable, unfavorable and linear if R_L_ = 0, 0 < R_L_ < 1, R_L_ > 1 and R_L_ = 1, respectively.

##### Freundlich Isotherm Model

A Freundlich isotherm model is used for describing the adsorption onto heterogeneous surfaces. It possesses the formation of multilayer adsorption with interaction between the adsorbate molecules. Moreover, the adsorption active sites are non-identical for the occupation of adsorbate due to differences in adsorption energy. Its linear form is given by Equation (14).

(14) $$\;\ln q_{e}=\ln K_{F}+\frac{1}{n}\ln C_{e}$$

where q_e_ is the adsorbed dye amount at equilibrium (mg g^−1^) and C_e_ is the equilibrium concentration of dye in the solution at a constant temperature (mg L^−1^). K_F_ is the adsorption capacity ((mg g^−1^) (L mg^−1^)^1/n^), while 1/n shows an indication of the favorability and feasibility of the adsorption process and the surface heterogeneity, which is related to adsorption intensity. The value of 1/n indicates the type of isotherm; irreversible (1/n = 0); favorable (0 < 1/n < 1), and unfavorable (1/n > 1). By plotting ln q_e_ against ln C_e_, the constant *K_F_* and the indictor 1/n can be obtained.

##### Temkin Isotherm Model

The Temkin model assumes the decrease in heat uptake for dye molecules with surface saturation under the effect of continuous interaction between the studied adsorbent and dye molecules. The Temkin model can be expressed by Equation (15).

q_e_ = B In k_T_ + B In C_e_ (15)

where B is the Temkin constant that is controlled by the adjusted uptake temperature (J mol^−1^), K_T_ is the binding constant of Temkin isotherm (L g^−1^), q_e_ is the amount of dye adsorbed at equilibrium (mg g^−1^), and C_e_ is the dye equilibrium concentration at a constant temperature (mg L^−1^). By plotting q_e_ against ln C_e_, the constants B and K_T_ can be obtained from the slope and the intercept, respectively.

##### Dubinin–Radushkevich (D–R) Isotherm

The Dubinin–Radushkevich model is used for estimating the free energy of adsorption and the characteristic porosity. This model can successfully help in determining the type of the adsorption process whether it is chemisorption or physisorption. The (D–R) isotherm model does not assume that the surface is homogenous; so, it is more general than the Langmuir isotherm. The D–R isotherm is given by Equation (16).

ln q_e_ = ln (X_m_) − βε^2^ (16)

where q_e_ is the amount of dye adsorbed at equilibrium (mg g^−1^), X_m_ is monolayer saturation capacity (mg g^−1^), β is the activity coefficient that is related to adsorption mean free energy (mol^2^ j^−2^) and ε is the Polanyi potential which is determined by Equation (17).

(17) $$\varepsilon =\mathrm{RT}\;\ln\;(1+\frac{1}{C_{e}})$$

where R is universal gas constant (8.314 J mol^−1^K^−1^) and T is the absolute temperature (K).

By plotting ln q_e_ versus ε^2^ using Equation (16), a straight line is obtained with a slope and intercept β and ln X_m_, respectively. The mean free energy, E, of adsorption (kJ mol^−1^) can be determined by Equation (18):

(18) $$E=\frac{1}{{\left(2\beta \right)}^{0.5}}$$

This means that the free energy can determine the type of adsorption; if 8 < E < 16 kJ mol^−1^ the process is chemisorption, while if it is less than 8 kJ mol^−1^ the process is physisorption.

#### 2.4.5. Thermodynamic Studies

Thermodynamic parameters provide in-depth information on inherent energetic changes associated with adsorption; therefore, these parameters should be accurately evaluated. Studying thermodynamic parameters give a better understanding of the adsorptive behavior for the dye towards adsorbents. Gibbs free energy change, ∆G° (kJ mol^−1^), enthalpy change, ∆H° (kJ mol^−1^) and entropy change, ∆S° (J mol^−1^ K^−1^) were calculated for the adsorption of CR dye using four different temperatures (298, 308, 318 and 328 K) according to Equations (19) and (20).

∆G° = −RT ln K_c_ (19)

(20) $$\ln k_{c}=\frac{\Delta s{}^{\circ}}{R}-\frac{\Delta H{}^{\circ}}{\mathrm{RT}}$$

where R is the universal gas constant (8.314 J mol^−1^ K^−1^), T is the absolute temperature (K) and K_c_ is the distribution coefficient (q_e_/C_e_). By plotting ln K_c_ versus 1/T, the thermodynamic parameters can be obtained.

#### 2.4.6. Activation Energy

The activation energy (E_a_) is determined by applying Arrhenius equation, which refers to the minimum energy for proceeding the reaction as shown in Equation (21).

(21) $$\ln\;k=\ln\;A-\frac{E_{a}}{\mathrm{RT}}$$

where A is the Arrhenius factor, R is the universal gas constant (8.314 J mol^−1^ K^−1^), E_a_ is the activation energy (kJ mol^−1^) and T is the absolute temperature (K). By plotting ln k of the pseudo-second order constant versus inverse temperature (1/T), a straight line is obtained with slope of –E_a_/R, where E_a_ can be determined.

#### 2.4.7. Desorption Study

The dye desorption from adsorbent was performed by its washing with distilled water to remove any un-adsorbed dye molecules. Afterwards, the adsorbent (0.01 g) was immersed in the desorption medium (10 mL of ethanol, methanol, acetone or aqueous NaOH solution (0.1 N)) at 25 °C for 24 h. The amount of the desorbed dye can be calculated using Equation (22).

(22) $$\%\;\mathrm{Dye}\;\mathrm{desorption}\;={\;q}_{d}/q_{a}\times 100$$

where q_d_ is the amount of desorbed dye from the adsorbent surface (mg g^−1^) and q_a_ is the amount of the dye adsorbed onto the adsorbent (mg g^−1^) [24].

## 3. Results and Discussion

### 3.1. Synthesis of Novel CCs Adsorbent

The CCs adsorbent (Scheme 1) was prepared via a four-step procedure since the primary amine groups in chitosan were firstly protected by reaction with benzaldehyde for achieving chitosan Schiff’s base, in which the primary hydroxyl groups on C6 reacted with epichlorohydrin for generating epoxy chitosan Schiff’s base, followed by reaction of the epoxy rings with cyanoguanidine for attaining cyanoguanidine chitosan Schiff’s base, which was finally hydrolyzed in acidic medium to eliminate the benzaldehyde moieties and retrieve the amino groups to get CCs adsorbent. The amino and hydroxyl groups on chitosan in addition to the basic functional groups of cyanoguanidine incorporated into chitosan can potentially remove the acidic pollutants such as acidic dyes.

### 3.2. Characterization of Novel CCs Adsorbent

#### 3.2.1. FTIR Spectra of CCs Adsorbent

**Figure 2:**
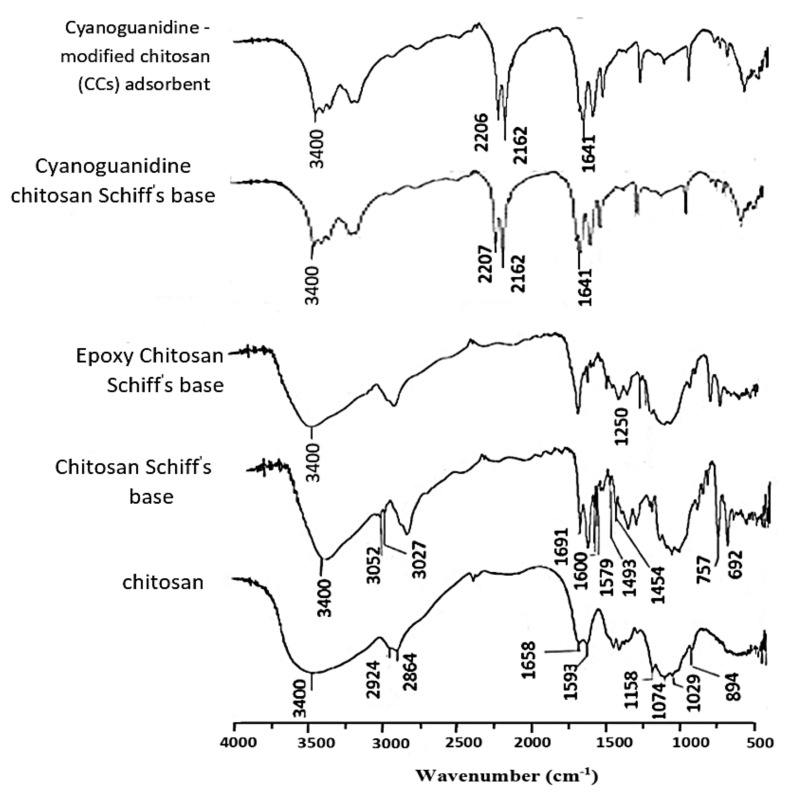
FTIR spectra of the modified chitosan derivatives.

FTIR spectra of chitosan and its modified derivatives were demonstrated in Figure 2. In the spectrum of chitosan, the existence of the saccharide moieties was confirmed by the appearance of four absorption peaks at 1158, 1074, 1029, and 894 cm^−1^. A dense broad absorption peak at around 3700 to 3000 cm^−1^ appeared, relating to the stretching vibration of -OH groups overlapped with that for -NH_2_ and their hydrogen bonds. The symmetric absorption peak corresponded to -CH and -CH_2_ groups in the pyranose rings appeared at 2924 and 2864 cm^−1^, respectively. The high extent of deacetylation of chitosan was confirmed by the appearance of two weak absorption peaks at 1658 and 1593 cm^−1^ assigning to amide I and amide II, respectively. The overlapping between the amino groups deforming vibration at 1600 cm^−1^ and the stretching vibration peak of amide I at 1658 cm^−1^ resulted in an intensive peak [22,26].

The spectrum of chitosan Schiff’s base displayed similar absorption peaks of chitosan in addition to some new peaks as follows: (1) at 3052 and 3027 cm^−1^ indicated to C-H groups in aromatic ring, (2) at 1691 cm^−1^ corresponded to C=N groups, (3) at 1600, 1579, 1493 and 1454 cm^−1^ related to C=C bond in aromatic rings, and (4) at 757 and 692 cm^−1^ (strong) due to mono-substituted benzene rings [16,21].

Epoxy chitosan Schiff’s base spectrum, in addition to the afore-mentioned peaks, displayed a new peak at 1250 cm^−1^ due to the epoxide moieties [27]. The spectrum of the cyanoguanidine chitosan Schiff’s base showed that the disappearance of the peak corresponded to the epoxide linkages at 1250 cm^−1^ and the appearance of two new peaks at 2207 and 2162 cm^−1^ related to C≡N group of the cyanoguanidine moiety [28], indicating occurrence of the interaction between the epoxide rings and the NH_2_ groups of cyanoguanidine. Moreover, the stretching vibration peak corresponded to C=N group of the cyanoguanidine moiety appeared at 1641 cm^−1^.

Removal of benzaldehyde moieties to obtain CCs adsorbent was confirmed by the disappearance of the absorption bands of mono-substituted benzene ring at 757 and 692 cm^−1^.

#### 3.2.2. Powder X-ray Diffraction of CCs Adsorbent

**Figure 3:**
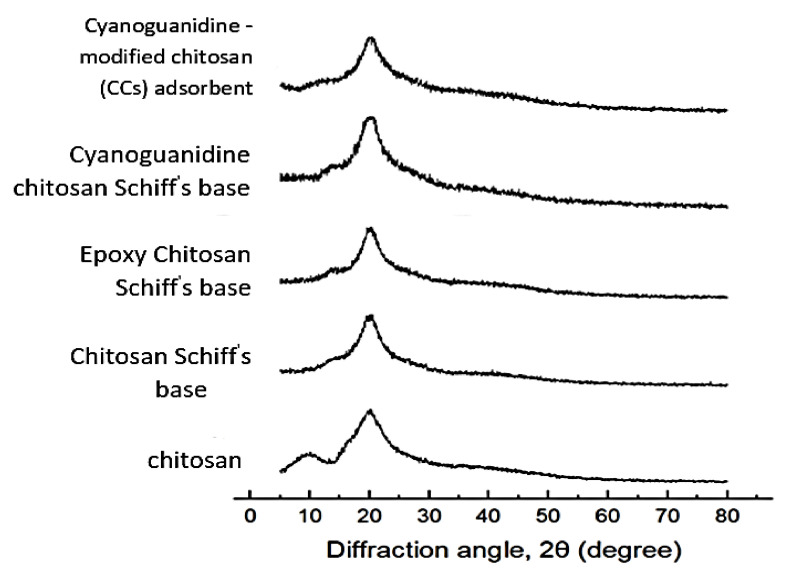
X-ray diffraction patterns of the modified chitosan derivatives.

X-ray diffractometry was used to explore the inner structures of the modified chitosan derivatives and their X-ray diffraction patterns were shown in Figure 3. There were two broad peaks in chitosan appeared near to 2θ = 10° and 20° which were attributable to its amorphous and crystalline regions, respectively [29]. This can be ascribed to the formation of the hydrogen bonds along its chains due to its possession of a great number of hydroxyl and amino groups. On the other hand, the modified chitosan derivatives were less crystalline than chitosan. This was illustrated by a disappearance of the peak at 2θ = 10° and a decrease of the intensity of the peak at 2θ = 20°. After modification of chitosan, its functionality greatly changed with a reduction of the hydrogen bonds due to consumption of its polar –NH_2_ and/or –OH groups and incorporation of the modifiers’ moieties that separated the chitosan chains away from each other. This led to an increase of the amorphous region and a decrease of the crystalline region.

#### 3.2.3. SEM Analysis of CCs Adsorbent

**Figure 4:**
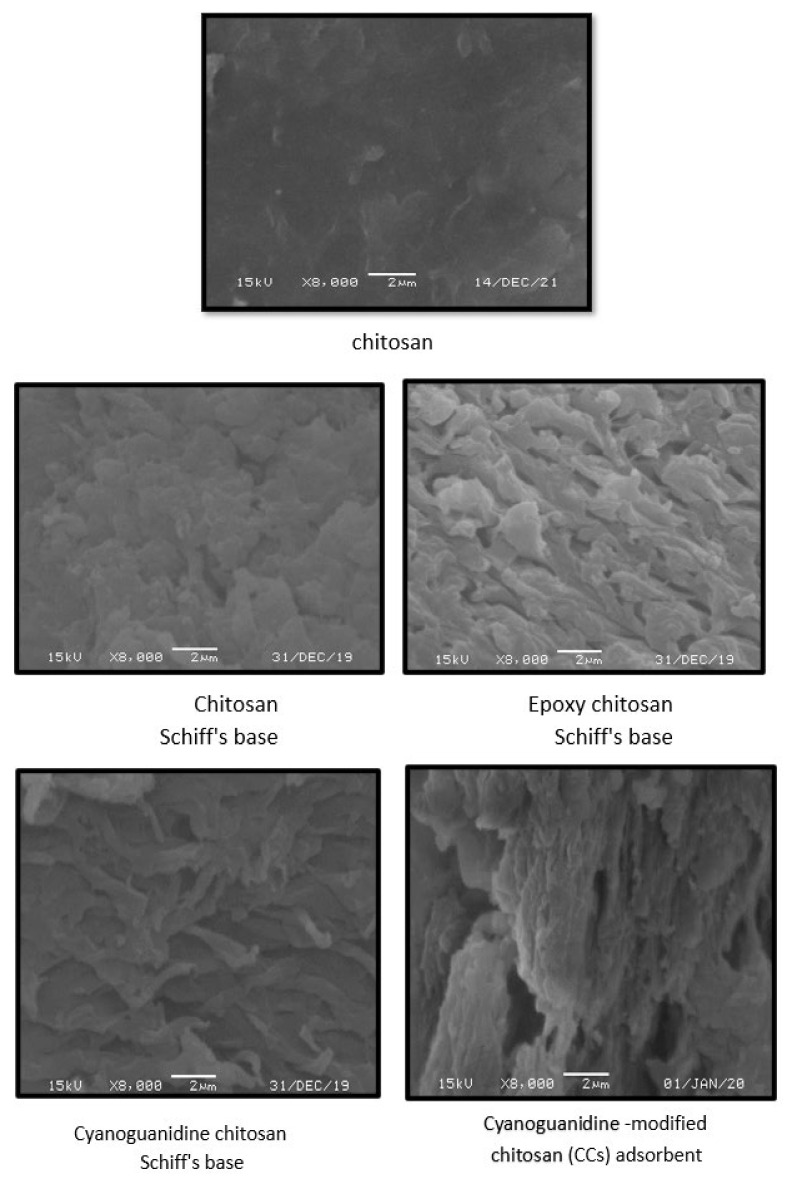
SEM images of the modified chitosan derivatives.

SEM micrographs of surface topography of the modified chitosan derivatives were shown in Figure 4. The original chitosan showed a smooth surface; however, its derivatives showed a raucous surface, containing lumps of diverse sizes because the incorporated substituent groups have assorted sizes. It can be noted that the lumps were homogeneously distributed over each derivative, suggesting that every stage for the chitosan modification process was successfully completed. The inserted substituent groups separated chitosan chains away from each other, reduced the formation of their hydrogen bonds and created a porous matrix with a large surface area.

### 3.3. Adsorption of CR Dye Using CCs Adsorbent

CCs was used in the present work for the removal of CR dye from water. Its adsorption capacity came from the incorporation of functional chelating cyanoguanidine moieties, which possessed more binding centers for CR dye. Various adsorption factors were studied, in addition to studying kinetics, isotherm and thermodynamics for understanding the adsorption mechanism.

#### 3.3.1. Optimization of the Adsorption Conditions

##### Effect of pH

One of the main parameters that has a major role on adsorption of dyes is the pH of the solution due to some mechanisms such as protonation processes between dyes and adsorbent surface.

**Figure 5:**
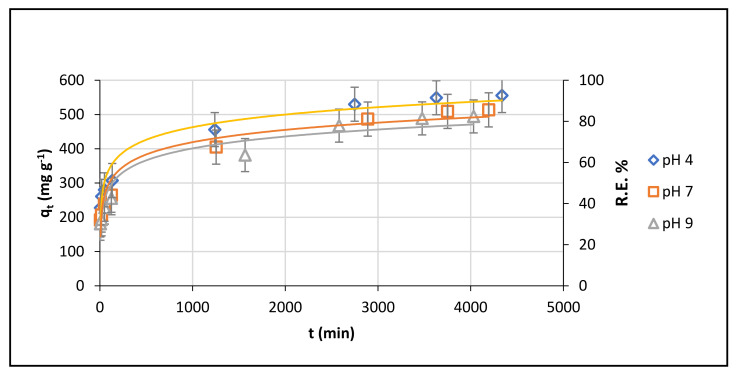
Effect of pH on CR dye adsorption onto CCs (Experimental conditions: 50 mg of adsorbent, 50 mL of dye solution (600 mg L^−1^) and temperature 55 °C).

The effect of pH on the adsorption of CR dye onto CCs adsorbent was studied at different pH values of 4, 7 and 9, and the results were presented in Figure 5. It can be noted that the adsorption of CR dye onto CCs decreased with increasing pH values. From the results, the percentages of CR dye removal were 92.55, 85.59 and 82.40% at pH 4, 7 and 9, respectively. This is in agreement with the study for the removal of CR dye using chitosan-coated quartz sand [30].

At low pH, an electrostatic attraction between the positively charged active sites on CCs adsorbent and dye anions takes place. The incorporation of cyanoguanidine moieties improved the adsorption performance of CCs, because they introduced more positive charges onto the CCs surface [31].

At a high pH, a strong competition between OH^−^ groups and dye anions for the positively charged sites of CCs occurred. Since the OH^−^ groups have a smaller size than the dye anions, binding of OH^−^ groups with CCs predominated, decreasing the adsorption performance of CCs.

Interestingly, the cyanoguanidine moieties in CCs have an important role in enhancing and improving its adsorptive capacity. The % R.E. values of CR dye (adsorbent dose = 50 mg, dye solution (50 mL, 600 mg L^−1^) pH = 9 and temperature = 55 °C) by chitosan Schiff’s base, epoxy chitosan Schiff’s base and cyanoguanidine chitosan Schiff’s base were 20.66, 23.70 and 41.48%, respectively, which were lower than that of CCs (82.40%).

##### Effect of Temperature

**Figure 6:**
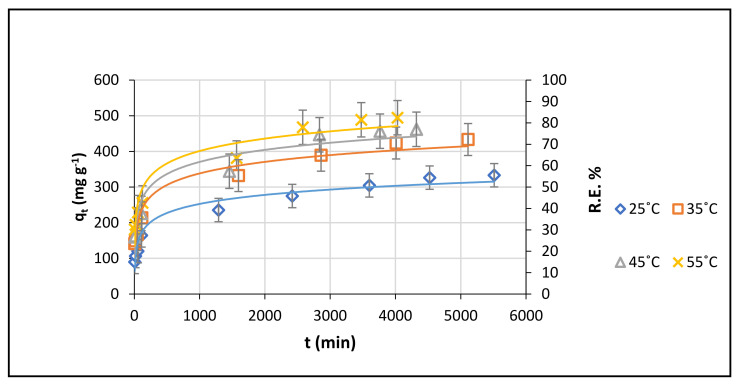
Impact of temperature on adsorption of CR dye onto CCs (Experimental conditions: 50 mg of adsorbent, 50 mL of dye solution (600 mg L^−1^) and pH = 9).

Temperature is an important parameter which can change the adsorption capacity of adsorbent and highly affect the adsorption of dyes from aqueous solution. The adsorption of CR dye onto CCs was studied at four different temperatures (25, 35, 45 and 55 °C) and the results were illustrated in Figure 6. It was obvious that increasing the temperature was accompanied by increasing the adsorption capacity of the adsorbent towards CR dye molecules. The % R.E. values were 55.51, 72.22, 77.0 and 82.40% at temperatures 25, 35, 45 and 55 °C, respectively. This indicated that a high temperature can facilitate the adsorption of CR dye onto CCs. This might be attributed to the swelling effect of the internal surface of an adsorbent, which contributed for the penetration of dye molecules into the interlayer of the adsorbent. This indicated that the adsorption of CR dye onto CCs was endothermic in nature [32]. Additionally, the gradual increase of temperature was followed by an increase in dye diffusivity and an increase in the dimensions of adsorbent pores. This consequently led to a reduction in the contribution of intraparticle resistance and decreased the effect of the boundary-layer [30]. The maximum adsorption capacity was achieved at 55 °C, which was used as the optimum temperature for batch experiments.

##### Impact of Initial Concentrations of the Dye

The adsorbed dye amount is strongly dependent on the initial dye concentration. This finding comes from the relationship between the initial dye concentration and the available active sites onto the adsorbent surface. In the present study, the use of 150 mg L^−1^ dye solution resulted in a complete adsorption, which reached 100% of CR dye removal (Experimental condition: pH = 9, temperature = 55 °C and equilibrium time = 6 h).

**Figure 7:**
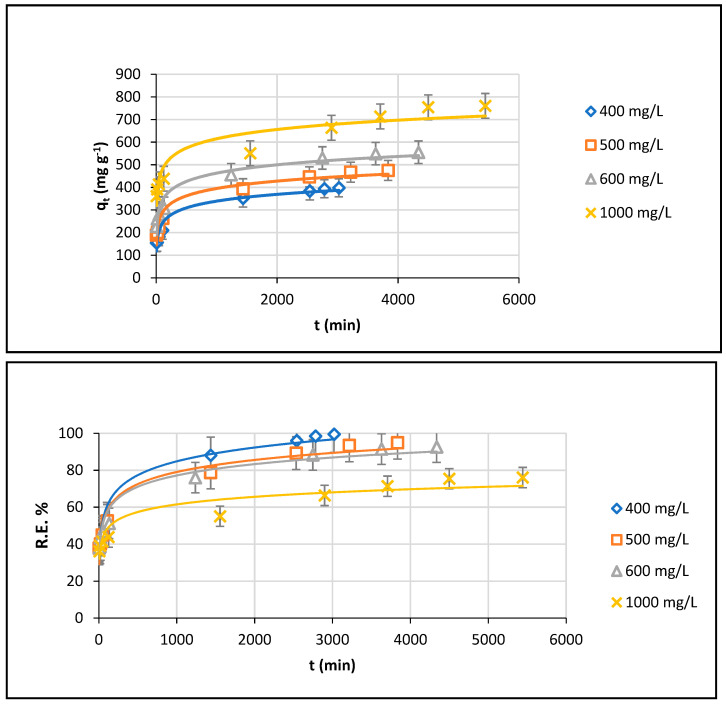
Impact of concentration of CR dye on adsorption onto CCs (Experimental conditions: 50 mg of adsorbent, 50 mL of dye solution, temperature 55 °C and pH = 4).

The adsorption of CR dye using CCs was studied at different concentrations (400, 500, 600 and 1000 mg L^−1^) and the results were illustrated in Figure 7. It could be noted that increasing the initial concentration of the dye was accompanied by a decrease in the removal percentage of CR dye. It decreased gradually to 99.5, 94.9, 92.55 and 76% at concentrations 400, 500, 600 and 1000 mg L^−1^, respectively. This can be attributed to the limited obtainable reactive centers available for adsorption process at higher concentrations. While the adsorption capability increased with increasing initial dye concentration, this is attributed to the high driving force of the concentration gradient at higher initial CR dye concentrations [30]. The maximum removal rate of CR dye was achieved at initial dye concentration of 400 mg L^−1^, which was considered the optimum concentration.

#### 3.3.2. Adsorption Kinetics

Studying adsorption kinetics is essential for suggesting the mechanism of adsorption, determining the optimum parameters for reaching the maximum removal percentage. and explaining the rate of dye uptake. The effect of time on the adsorption of CR dye using CCs was studied at different pHs, temperatures and initial dye concentrations using three kinetic models: pseudo-first-order, Equation (6), pseudo-second-order, Equation (8), and Elovich model, Equation (9), in order to understand the adsorption mechanism.

##### At a Different pH

**Figure 8:**
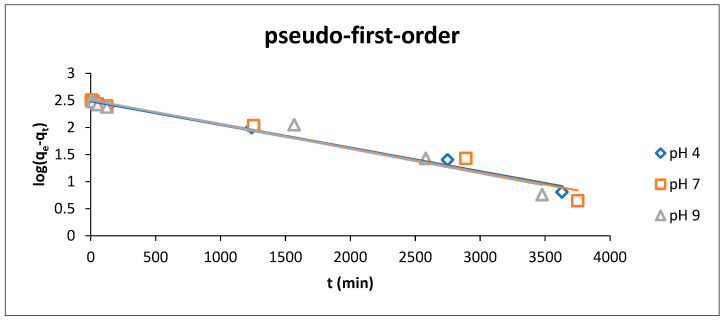

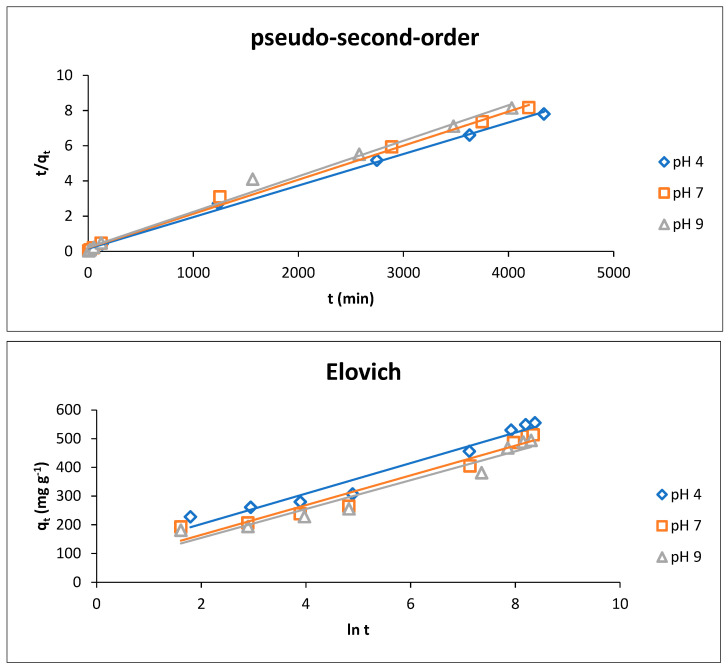
Linear regressions of kinetic plots at different pH for the adsorption of CR dye onto CCs.

The results of kinetic data of the adsorption of CR dye onto CCs are summarized in Table 1. The plots of the adsorption for the three kinetic models at pH 4, 7 and 9 were illustrated in Figure 8.

The pseudo-second-order model showed the highest value of correlation coefficient (R^2^) compared with the other models. It ranged from 0.997 to 0.991 at pH values from 4 to 9. This indicated that the pseudo-second order model has a perfect fit to describe the CR dye adsorption by CCs surface. In addition, there is a good agreement between the values of experimental and calculated q_e_ for the pseudo-second-order, which is equal to 555.33 and 555.56 mg g^−1^, respectively, at pH = 4. This reflected and confirmed the excellent fitting of the pseudo-second-order kinetic model for the adsorption process of CR dye onto CCs.

On the other hand, the values of R^2^ obtained the for pseudo-first order showed relatively lower values than that obtained by the pseudo-second order model, since they ranged from 0.989 to 0.958 at pH values from 4 to 9. Additionally, the theoretical equilibrium adsorption capacities, q_e_, for pseudo-first-order were significantly different from that of the experimental ones which equal to 555.33 and 310.38 mg g^−1^, respectively, suggesting the inadequacy of the pseudo-first-order model for describing the adsorption kinetics of CR dye onto CCs.

The normalized standard deviation, Δq_e_, (%) was applied to check the validity of the pseudo-first-order and pseudo-second-order models quantitatively. Based on the values of Δq_e_ (%) shown in Table 1, it is clearly noted that the pseudo-second-order model showed the best fit since it yielded lower values of Δq_e_ (%) than those obtained from pseudo-first-order at different pH values. These results confirmed that the adsorption kinetic studies of CR dye onto CCs would be more favorable when applied by pseudo-second-order. Comparable data were published in a previous study to remove CR dye using chitosan-coated quartz sand [30].

The fitness of the pseudo-second order kinetic model for the adsorption of CR dye onto CCs indicated that the adsorption process was proceeded by various mechanisms, including chemical interaction and electrostatic attraction of anions of the dye with the binding reactive centers onto the surface of CCs [30].

On the other hand, it is noted that the values of rate constant, *k*_2_, decreased gradually with the increase in the pH from 4 to 9, suggesting that pH 4 was the optimum value for adsorption. At pH 4, an enhancement in the electrostatic attraction force between the protonated sites of CCs surface and the negatively charged sulfonate groups (–SO_3_^−^) of CR dye molecules took place. This led to an increase in the adsorption process at a low pH.

The Elovich model is one of the most interesting kinetic models that has a wide applicability to describe the adsorption systems related to the chemical nature. This model occurs when the adsorption involves the chemisorption reaction onto adsorbent surface, and the adsorption speed decreases as time passes due to covering of the adsorbent surface with an adsorbate [33].

Figure 8 showed the linear plots of the Elovich kinetic model, which was obtained by applying Equation (9) at pH 4, 7 and 9. The values of α, β and the respective R^2^ were summarized in Table 1. The obtained correlation coefficients were relatively high, which confirmed that this model fitted fully to the experimental results of the adsorbent. The values of R^2^ ranged from 0.96 and 0.94 at pH from 4 to 9. The good fitness of the experimental data for Elovich confirmed that the adsorption of CR dye onto CCs was controlled by a chemisorption process.

On the other hand, it was obvious that the values of the initial adsorption rate, α, decreased with increasing pH values. They ranged from 325.64 to 148.17 mg g^−1^ min^−1^ at pH values from 4 to 9 for the adsorption of CR dye onto CCs. Whereas, the values of the extent of surface coverage, β, increased with increasing pH; they ranged from 0.019 to 0.020 g mg^−1^ at pH values from 4 to 9.

##### At Different Temperatures

**Figure 9:**
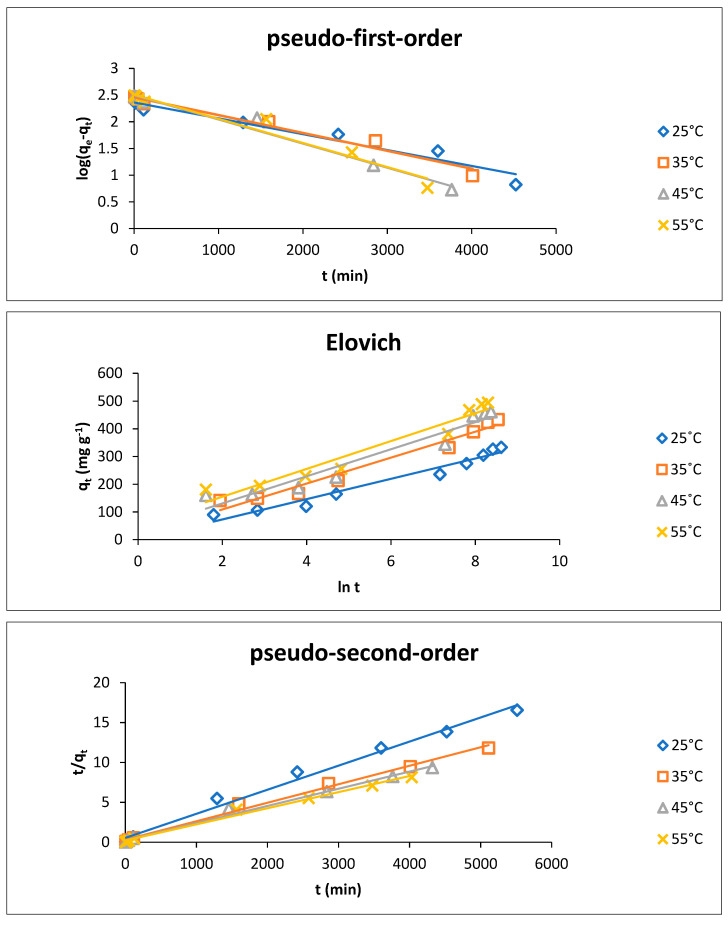
Linear regressions of kinetic plots at different temperatures for the adsorption of CR dye onto CCs.

Temperature is a crucial parameter which highly affects both adsorption performance and adsorbent behavior. The experimental results of kinetic data were summarized in Table 2 for the adsorption of CR dye onto CCs. The plots showing the three kinetic models for CR dye adsorption by CCs, at 25 to 55 °C were illustrated in Figure 9.

Based on the high values of correlation coefficient (R^2^) and the low values of the normalized standard deviation, Δq_e_ (%), it was clearly apparent that the pseudo-second-order model showed fully fit for the obtained results compared with pseudo-first-order model. R^2^ values obtained from pseudo-second-order adsorption model ranged from 0.990 to 0.991 at 25 to 55 °C. In addition, the q_e_ for pseudo-second-order model was similar to that of the experimental one, since the values of experimental and calculated q_e_ for pseudo-second-order model at 55 °C equal to 494.44 and 500 mg g^−1^, respectively. On the other hand, the correlation coefficient obtained from the pseudo-first order model showed lower values than those obtained from pseudo-second order, since they ranged from 0.957 and 0.958 at temperatures from 25 and 55 °C for the adsorption of CR dye onto CCs. In addition, the experimental and calculated capacities showed different values and equalled 494.44 and 322.18 mg g^−1^ at 55 °C, respectively. This indicated the unsuitability of the pseudo-first order model for describing the adsorption process.

Additionally, the value of rate constant, k_2_, increased gradually by increasing the temperature, as it increased from 1.64 × 10^−5^ to 1.81 × 10^−5^ g mg^−1^ min^−1^ when the temperature increased from 25 to 55 °C. This can be attributed to the low viscosity of the dye solution, which subsequently entrapped more dye molecules onto the adsorbent surface [34]. Furthermore, this might be due to the increase in collision probability between the active sites of adsorbent and the adsorbate and the decrease in the boundary layer thickness of adsorbent at an elevated temperature [35]. In addition, this might be due to the effect of swelling of the adsorbent internal structure, resulting from increasing temperature. This led to a greater diffusion capability for dye molecules into the adsorbent surface, reaching to its internal structure, and finally increasing the rate of adsorption [32].

Figure 9 showed the linear form of the Elovich model (Equation (9)). The values of the initial adsorption rate constant (α) and the desorption constant (β) at different temperatures (25, 35, 45 and 55 °C) were summarized in Table 2. Obviously, the experimental data for the adsorption of CR dye onto CCs agreed with the Elovich model. This result was reflected by R^2^ values which ranged from 0.963 to 0.940 at temperatures from 25 to 55 °C. The Elovich model assumes that the adsorbent active sites were heterogenous, and exhibited various energies for chemical adsorption [33]. This model is used for describing the kinetics of ion exchange systems. Hence, it is concluded that the adsorption of CR dye onto CCs was chemisorption. This finding is in agreement with the above results observed throughout the present study, which concluded that pseudo-second order is the best fit model. The obtained results also suggested that the chemisorption is the rate-determining step for the adsorption of CR dye onto CCs.

On the other hand, based on the results in Table 2, the values of initial adsorption rate constant, α, showed an increase with the increasing temperature. It ranged from 36.24 to 148.17 mg g^−1^ min^−1^ when the temperature increased from 25 to 55 °C for the adsorption onto CCs, whereas the desorption constant, β, showed a decrease with increasing temperature; the values of β ranged from 0.027 to 0.020 g mg^−1^ when the temperature increased from 25 to 55 °C for the adsorption onto CCs. This is attributed to the low number of available binding sites for the adsorption at an elevated temperature [36].

##### At Different Initial Dye Concentrations

**Figure 10:**
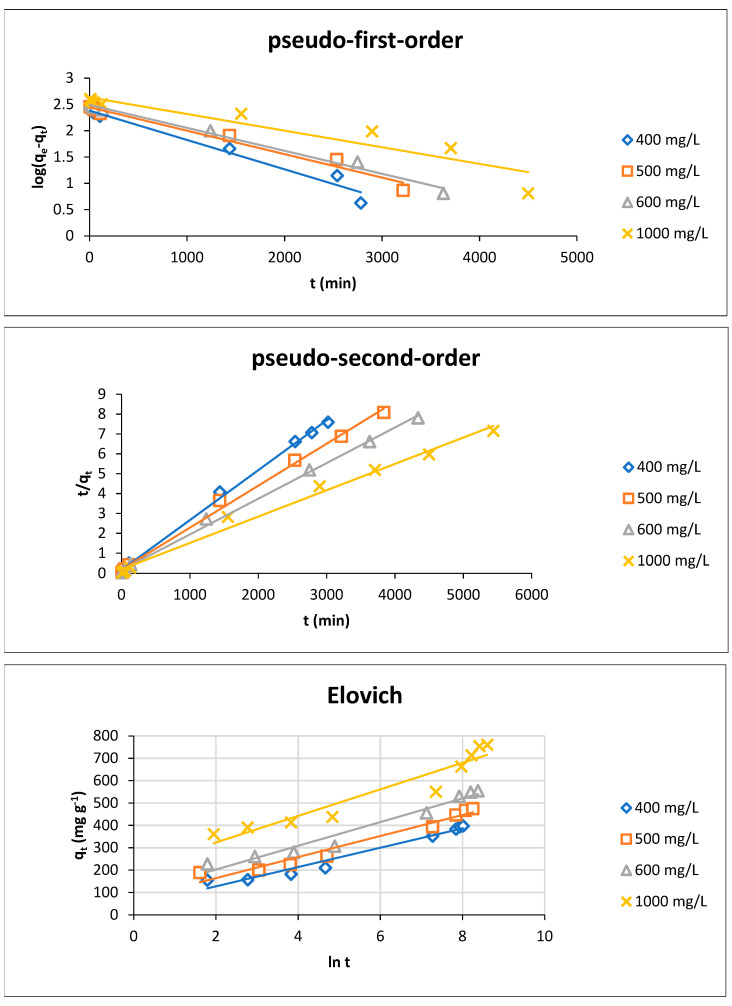
Linear regressions of kinetic plots for the adsorption of CR dye onto CCs.

The experimental kinetic data were summarized in Table 3. Figure 10 showed the graphical linear forms of the studied three models of kinetics for adsorbing CR dye onto CCs at different initial dye concentrations (400, 500, 600 and 1000 mg L^−1^).

The adsorption of CR dye onto the adsorbent showed excellent compliance with the pseudo-second-order, better than the pseudo-first-order, according to their respective correlation coefficient (R^2^) values and the values of the normalized standard deviation, Δq_e_ (%). The R^2^ values obtained for pseudo-second-order ranged from 0.998–0.990, which were higher than those obtained from the pseudo-first-order (0.972–0.879) at concentrations which ranged from 400–1000 mg L^−1^. On the other hand, the values of the normalized standard deviation, Δq_e_ (%), showed lower values for the pseudo-second-order than the pseudo-first-order. In addition, the calculated equilibrium capacities were close to the experimental ones for the pseudo-second-order. These findings indicated that the adsorption kinetics of CR dye by CCs perfectly followed the pseudo-second-order model, implying that the overall rate of the adsorption process was controlled by chemisorption [35]. Similar results were obtained for adsorption of CR dye onto trimellitic anhydride isothiocyanate-cross-linked chitosan hydrogels [23].

Regarding the values of rate constants for pseudo-second-order model, k_2_, there was a significant decrease for k_2_ values with increasing dye concentrations, as it decreased from 3.97 × 10^−5^ to 0.88 × 10^−5^ g mg^−1^ min^−1^ when the dye concentration increased from 400 to 1000 mg L^−1^. The same results are in agreement with another study [36]. The obtained results confirmed that the optimum concentration value was 400 mg L^−1^. This might be ascribed to the decrease in the unoccupied available reactive centers with time, which led to a decrease in the adsorption rate [35].

The linear form of Elovich model, represented in Equation (9), was illustrated in Figure 10 at different dye concentrations (400–1000 mg L^−1^) for the adsorption onto CCs. The values of the initial adsorption rate, α, and the extent of surface coverage, β, were summarized in Table 3. It is noted that the experimental results agreed adequately with the Elovich model, since R^2^ ranged from 0.963 and 0.913 at a dye concentration from 400 and 1000 mg L^−1^. The good applicability of the Elovich model indicated that the process was governed by chemisorption. On the other hand, the values of the initial rate of adsorption increased with increasing concentrations, and it increased from 111.97 to 1874.60 mg g^−1^ min^−1^ by increasing concentrations from 400 to 1000 mg L^−1^. This result might be ascribed to the higher concentration gradient, whereas a decrease in the extent of coverage (β) with increasing concentration was observed and decreased from 0.23 to 0.017 g mg^−1^ with increasing concentrations from 400 to 1000 mg L^−1^. Since at a higher concentration the gradient forces the particles of CR dye to show more adsorption towards the surface of adsorbent, this might negatively affect the desorption process [37].

#### 3.3.3. Mechanism of CR Dye Adsorption onto CCs

Mainly, in the aqueous solution, the CR dye (CR-SO_3_Na) would dissolve and dissociate giving the dye anions (CR-SO_3_^−^) as shown in Equation (23).

CR-SO_3_Na→CR-SO_3_^−^ + Na^+^ (23)

CCs is characterized by polar functional groups (–NH_2_ and –OH) on its surface. At a low pH, the protonation of these groups took place, leading to the formation of NH_3_^+^ and -OH_2_^+^, respectively. Thus, an electrostatic interaction occurred between these two positively charged functional groups with the negatively charged dye anions, as shown in Equations (24) and (25).

(24) $$-\mathrm{OH}\;\overset{H^{+}}{\to }-{\mathrm{OH}}_{2}^{+}\overset{\mathrm{CR}-{\mathrm{SO}}_{3}^{-}}{\to }\mathrm{CR}-{\mathrm{SO}}_{3}^{-}{\mathrm{OH}}_{2}^{+}-$$

(25) $$-{\mathrm{NH}}_{2}\overset{H^{+}}{\to }-{\mathrm{NH}}_{3}^{+}\overset{\mathrm{CR}-{\mathrm{SO}}_{3}^{-}\;}{\to }\;\mathrm{CR}-{\mathrm{SO}}_{3}^{-}{\mathrm{NH}}_{3}^{+}-$$

**Scheme 2:**
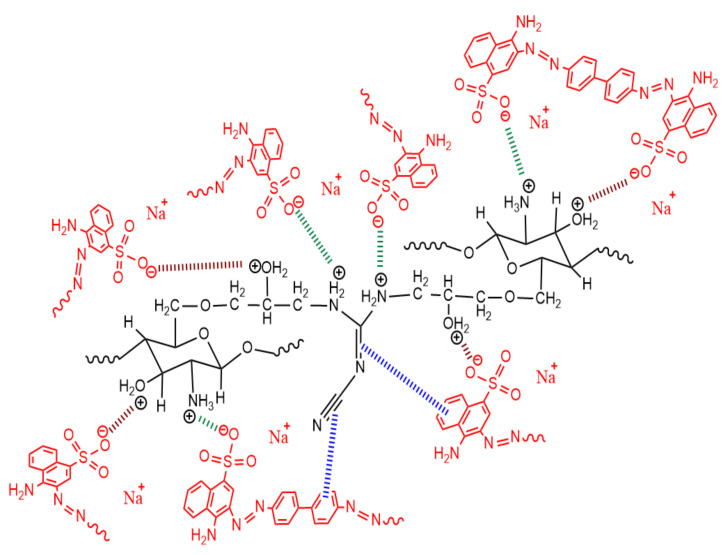
Schematic illustration of the interaction of CR dye with CCs at a low pH.

Additionally, the surface of CCs contains several heteroatoms, comprising oxygen and nitrogen. Therefore, binding of CR dye on the CC surface via hydrogen bonding and Van der Waals forces cannot be ruled out [30]. Thus, the possible mechanism involves a combination of adsorbent–sorbate electrostatic interactions, between the positively charged protonated groups and the negatively charged CR dye ions, in addition to H-bonding interactions and other physical forces such as π–π stacking and van der Waals forces [38]. Whereas, the electrostatic interaction might be the main mechanism for the removal behavior (Scheme 2).

To identify and explore the diffusion mechanism for the adsorption of the CR dye, the intraparticle diffusion model was applied. By studying the intraparticle diffusion, the adsorption process involves a multi-step process: (i) bulk diffusion of dye molecules, (ii) film diffusion; dye molecules can diffuse to the surface of adsorbent through the boundary layer, (iii) intraparticle diffusion; dye molecules are transported from the surface into adsorbent pores, and (iv) dyes are adsorbed onto the adsorbent active sites through chemical or physical reaction. Due to the continuous stirring of the batch system, ignoring diffusion by bulk could be suggested and the step that determines the rate would be the intraparticle diffusion in biosorption [39].

##### The Intraparticle Diffusion

The intraparticle diffusion model was studied for the determination of the rate determining step. When the plot of qt versus t^1/2^ (Equation (10)) gives a straight-line passing via the origin, this implies that the process of adsorption is governed by diffusion, and the particle diffusion would be the rate controlling step. However, if the plot shows two or more linear regions, this indicates that the adsorption process takes place by a multistage adsorption [36].

**Figure 11:**
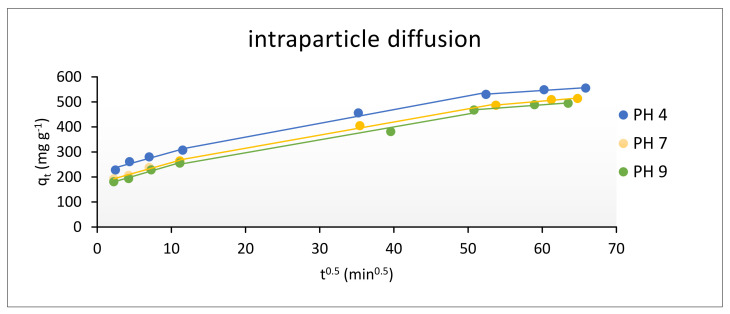
The intraparticle diffusion plots for adsorption of CR dye onto CCs using different pH values.

**Figure 12:**
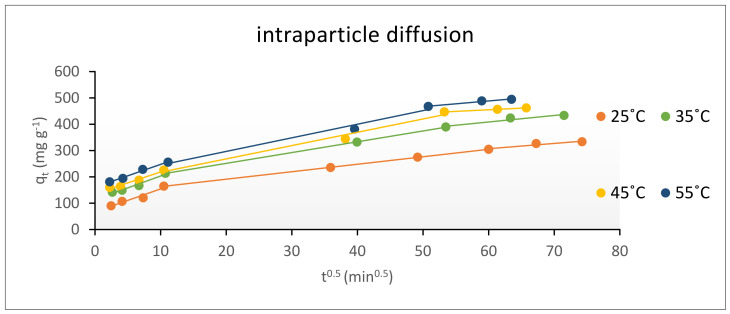
The intraparticle diffusion plots for adsorption of CR dye onto CCs at different temperatures.

**Figure 13:**
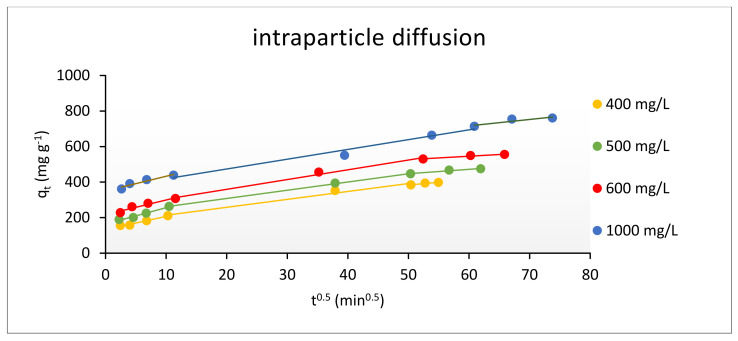
The intraparticle diffusion plots for adsorption of CR dye onto CCs using different initial concentrations.

The intraparticle diffusion results for the adsorption of CR dye onto CCs were analyzed at different pH values (from 4 to 9), temperatures (from 25 to 55 °C) and initial dye concentrations (from 400 to 1000 mg L^−1^), and are illustrated in Figure 11, Figure 12 and Figure 13, respectively. The obtained plots did not pass through the origin. This confirmed that the intraparticle diffusion model was not the only rate-limiting step, suggesting other kinetic factors controlling and contributing the rate and the mechanism of adsorption of CR dye onto CCs. The multilinearity of the curves confirmed that the adsorption of CR dye molecules into the adsorbent surface took place in three stages. Firstly, the dye molecule diffused into the external surface of adsorbent by film diffusion, then in a slower step it diffused into the internal pores of the adsorbent throughout the intraparticle diffusion. The second stage was more stable than the first stage, indicating that it was the step for determining the rate, and the final step corresponded to the stage for final equilibrium [40].

The first slope (k_int.1_) represented the fast adsorption of dye molecules onto adsorbent surface by electrostatic interactions, while the second slope (k_int.2_) revealed to the diffusion of dye molecules into the internal pores of adsorbent. The third slope (k_int.3_) corresponded to the saturation of all active sites on adsorbent surface (equilibrium stage). These findings suggested that CR dye adsorbed onto the surface of adsorbent and in its internal structure [38].

Studying the intraparticle diffusion with the variation of pH was carried out using pH values of 4, 7 and 9. The values of intraparticle diffusion rate (k_int_) and correlation coefficients (R^2^) were summarized in Table 4 for the adsorption of CR dye onto CCs. It is noted that the values of k_int.2_, decreased with increasing pH values; they equal 5.50, 5.24 and 5.17 at pH 4, 7 and 9, respectively. A similar result was illustrated in a previous study for adsorption of CR dye onto pine bark [41].

The intraparticle diffusion at 25, 35, 45 and 55 °C was studied, and k_int_ and R^2^ values were summarized in Table 5. The results showed an increase in the diffusion rate constant with the increasing temperature. For the adsorption of CR dye onto CCs, the diffusion rate constant (k_int.2_) increased from 2.84 to 5.17 mg g^−1^ min^−1^ when the temperature increased from 25 to 55 °C. All values of R^2^ are higher than 0.9, suggesting that the adsorption process took place by a combination mechanism.

The intraparticle diffusion was studied also at various initial dye concentrations (400, 500, 600 and 1000 mg L^−1^) and the values of intraparticle diffusion rate (k_int_) and correlation coefficients (R^2^) for adsorbing CR dye onto CCs were summarized in Table 6. It was found that the values of k_int_ increased with the increase in the concentration, since the values of k_int.2_ for the second stage equal to 4.46 and 5.51 mg g^−1^ min^−1^ at concentrations of 400 and 1000 mg L^−1^, respectively. These findings indicated that the diffusion of CR dye molecules into the interior pores of adsorbent surface increased with increasing the initial dye concentration, and it is in agreement with some previous studies [40].

#### 3.3.4. Adsorption Isotherm

The adsorption isotherm is considered one of the main important fundamentals for describing the mechanism of dye adsorption onto the adsorbent surface. These isotherms efficiently show the way of interaction between the dye and the sorbent surface.

**Figure 14:**
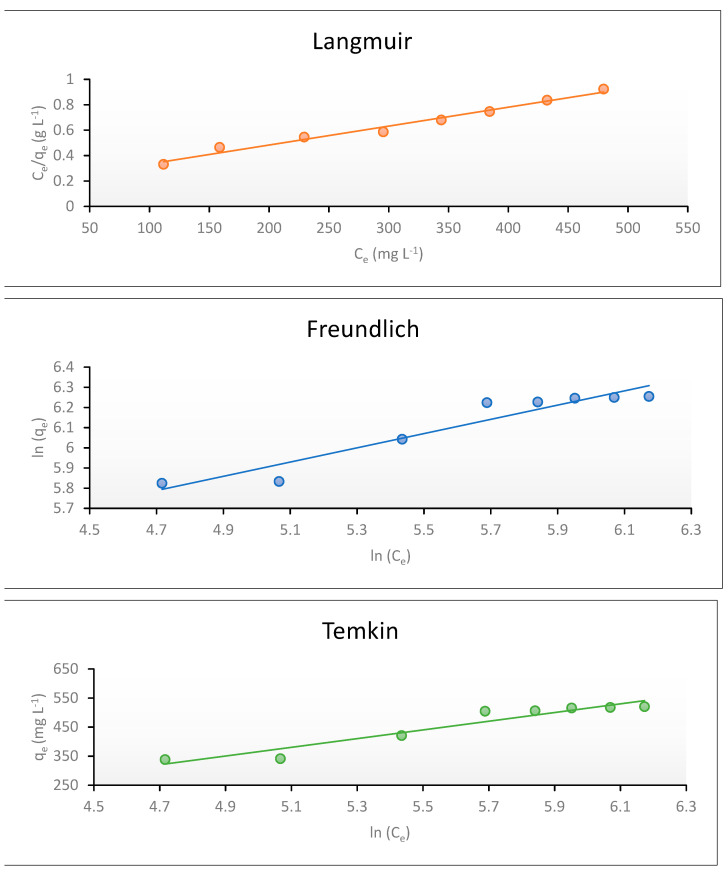

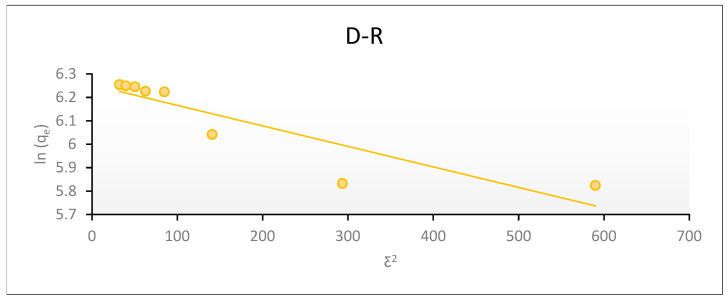
Adsorption isotherms for CR dye onto CCs at 55 °C and pH 9.

The adsorption isotherms for CR dye removal by CCs were represented in Figure 14 for Langmuir, Freundlich, Temkin and Dubinin–Radushkevich (D–R) isotherm models. Additionally, Table 7 listed the results of the four models and their corresponding fitting correlation coefficients (R^2^). The suitability of the model would be determined by the value of R^2^ which is the closest to unity [36].

According to R^2^ values, the adsorptive systems were better modeled by Langmuir adsorption isotherms than Freundlich adsorption isotherms, over the concentration ranges studied, since the value of R^2^ for Langmuir was 0.980 and for Freundlich was 0.917.

The good applicability of Langmuir adsorption isotherm for the removal of CR dye by the adsorbent indicated that the adsorption process occurred in a monolayer coverage manner with no interaction between adsorbed dye molecules with each other. In addition, the adsorption active sites were homogenously distributed on the adsorbent surface and they were identical for all dye molecules. Thus, each active site binds only with one dye molecule [39]. The maximum monolayer coverage capacity, q_max_, at 55 °C was 666.67 mg g^−1^ for the removal of CR dye using CCs. Previous studies also reported the Langmuir model as the best fit for the adsorption of dyes using chitosan [42]. The obtained R_L_ values for adsorbing CR dyes onto CCs ranged from 0.217 to 0.111, which indicates that the adsorption of CR dye onto CCs was a favorable process.

The Temkin model took into consideration the effect of the indirect interaction between the molecules of adsorbate. Assuming the linear decrease of adsorption heat of all the molecules in the layer due to the interactions between adsorbent and adsorbate, the adsorption process is characterized with a uniform distribution of binding energies [40].

Temkin constants, B_T_ and K_T_, were listed in Table 7. The obtained plot provided a curve which indicated that the adsorption process did not follow this model.

The Dubinin–Radushkevich (D-R) isotherm model is used to describe the nature of the adsorption process. It is similar to Langmuir, while it is more general because it rejects the homogenous surfaces [40]. The linear form of D-R isotherm (Equation (16)) was obtained by plotting lnq_e_ versus ε^2^, as illustrated in Figure 14. The values of β and ln X_m_ were calculated from the slope and intercept, respectively, and were listed in Table 7. Based on the value of R^2^, the D-R isotherm cannot be used to describe the adsorption of CR dye onto CCs, as it equals 0.807 for the adsorption onto CCs.

From the analysis of the experimental results showed in Table 7, it is concluded that the Langmuir isotherm model was the best model can fit the experimental results due to its highest R^2^. Freudlich and Temkin models did not show high accuracy as their correlation coefficients were not as high as the Langmuir isotherm, while the D-R model could not describe the adsorption process using CCs due to its low value of R^2^. These findings reflected the homogenous nature of the adsorbent. Therefore, it is assumed that the adsorption of CR dye by CCs took place uniformly onto its active sites [40].

#### 3.3.5. Adsorption Thermodynamics

The adsorption process of CR dye onto the adsorbent was studied using various temperatures (298, 308, 318 and 328 K). The values of thermodynamic parameters ΔG° were calculated according to Equation (19), while ΔH° and ΔS° were calculated from the Van’t Hoff linear plot (Equation (20)).

**Figure 15:**
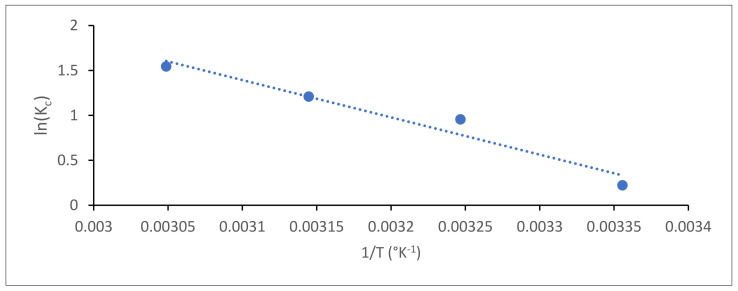
Adsorption thermodynamic of CR dye onto CCs.

The results were illustrated in Figure 15 and Table 8 for the adsorption CR dye using CCs. The values of ΔH° was 34.49 kJ mol^−1^, this positive value suggested that the interaction of CR dye adsorbed onto CCs was endothermic in nature [23].

The negative values of ΔG° at all selected temperatures indicated that the adsorption of CR dye onto CCs was a spontaneous process. It decreased with increasing temperature, and this indicated the feasibility and favorability of the process at elevated temperatures [32].

The positive value of ΔS° (118.48 J mol^−1^K^−1^) indicated that the randomness increased at the interface between adsorbent and adsorbate solution during CR dye adsorption by CCs [42]. It can be explained as follows: throughout the adsorption process, the dye molecule received more entropy due to the displacement of adsorbed solvent molecule which resulting finally in an increasing randomness [43]. This actually occurred due to increasing the number of free ions of adsorbate that were found in order form near adsorbent surface than the adsorbed ions before adsorption process. Hence, the distribution of rotational energy increased, which consequently increased randomness at the interface between solid and liquid, and finally resulted in an increasing adsorption [35]. These findings are in a good agreement with previous studies [42].

#### 3.3.6. Activation Energy

**Figure 16:**
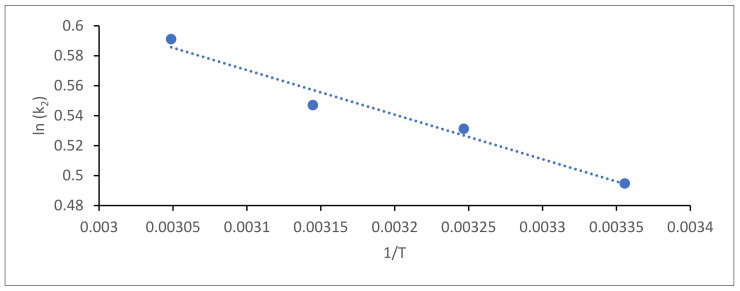
Arrhenius plot for adsorption of CR dye onto CCs.

The type of adsorption process, whether it is physisorption or chemisorption, can be determined by the magnitude of activation energy. When the activation energy is found in the range 0–40 kJ mol^−1^, this indicates that the reaction is physisorption, while if it is in the range 40–800 kJ mol^−1^, the reaction is chemisorption [24]. The value of E_a_ for the adsorption of CR dye onto CCs was 2.47 kJ mol^−1^, as illustrated in Figure 16. This value indicated that the adsorption was a physisorption in nature and was ascribed to a low potential barrier [44]. This result agrees with the adsorption of CR dye onto activated Moringa oleifera seed [45] and onto guava leaf-based activated carbon [36].

#### 3.3.7. Comparison between CCs and Other Adsorbents for CR Dye Removal

To evaluate the affinity and efficiency of the CCs adsorbent, it became of interest to compare its adsorption maximum capacity for removal of CR dye with other adsorbents in previous reported studies as shown in Table 9. It is clearly observed that CCs possesses the highest removal capacities for CR dye than other reported adsorbents. It could efficiently adsorb CR dye at 55 °C with q_max_ of 760.44 mg g^−1^. This proved that CCs is an efficient and a promising adsorbent for the removal of CR dye from aqueous solution.

#### 3.3.8. Desorption Studies

The reusability is considered one of the most important aspects to minimize the cost of adsorption by regeneration of adsorbent [51].

The desorption of CR dye was applied using Equation (22). No desorption was obtained by using ethanol, methanol and acetone as desorption medium, while using 0.1 N NaOH solution showed that the desorption percentage reached 58% after 5 cycles. These findings confirmed that CCs can be efficiently reused for the adsorption of CR dye from the aqueous solution.

## 4. Conclusions

Modification of chitosan was successfully done via a four-step procedure using cyanoguanidine at the final step. The produced cyanoguanidine-modified chitosan (CCs) possessed free primary amino and hydroxyl active centers in its main chains, in addition to nitrogen-rich cyanoguanidine as crosslinking linkages. Its structure was characterized by different techniques; FTIR, XRD and SEM. Its active basic groups acted as efficiently binding sites for the anionic CR dye from its aqueous solution. The adsorption capacity for CR dye onto CCs increased with increasing temperature reaching its maximum at 55 °C. The adsorption capacity decreased with increasing pH achieving its optimum value at pH 4. The removal percentage of CR dye decreased with the increase in the initial concentration of the dye. The pseudo-second-order model showed a perfect fit to describe the adsorption process, indicating the chemisorption process. The values of rate constant, k_2_, decreased gradually with the increasing pH from 4 to 9 and initial concentration of dye solution from 400 to 1000 mg L^−1^, while it increased with increasing temperature from 25 °C to 55 °C. The relatively high correlation coefficient R^2^ obtained from the Elovich model confirmed that this model fitted fully to the experimental results for the adsorbent at the studied parameters. This indicated that the adsorption of CR dye onto CCs was controlled by a chemisorption process. According to intraparticle diffusion, the transfer of CR dye molecules into the adsorbent surface took place in three stages. The plots of intraparticle diffusion showed that it was not the only rate-limiting step. The good applicability of the Langmuir adsorption isotherm indicated that the adsorption process occurred in a monolayer coverage manner with no interaction between adsorbed dye molecules with each other. The maximum monolayer coverage capacity, q_max_, at 55 °C was 666.67 mg g^−1^. The Freudlich and Temkin models did not show a high accuracy as their correlation coefficients were not as high as the Langmuir isotherm, whereas, the experimental data confirmed the unsuitability of the Dubinin–Radushkevich isotherm model. The positive value of ΔH° (34.49 kJ mol^−1^) suggests that the adsorption was endothermic in nature. The negative values of ΔG° at all selected temperatures reflected that the adsorption was a spontaneous process. The positive value of ΔS° indicated the randomness elevation at the interface between adsorbent and adsorbate solution. The value of activation energy E_a_ was 2.47 kJ mol^−1^, assigning that the adsorption was physisorption. Thus, it is concluded that the adsorption of CR dye CCs involved physisorption and chemisorption processes.

## Figures and Tables

**Table 1:** Kinetic model constants and correlation coefficients for the adsorption of CR dye onto CCs at different pH.

Kinetic Models	Parameters	PH		
		4	7	9
pseudo- first-order	q_e.exp_ (mg g^−1^)	555.33	513.56	494.44
	R^2^	0.989	0.969	0.958
	q_e.cal_ (mg g^−1^)	310.38	321.07	322.18
	K_1_ (10^−4^) (min^−1^)	9.21	9.21	11.15
	Δq_e_ (%)	16.67	14.17	13.17
pseudo- second-order	R^2^	0.997	0.996	0.991
	q_e,cal_ (mg g^−1^)	555.56	526.32	500
	K_2_ (10^−5^) (g·mg^−1^·min^−1^)	2.12	1.86	1.81
	Δq_e_ (%)	0.02	0.94	0.42
Elovich	R^2^	0.961	0.951	0.94
	β (g·mg^−1^)	0.019	0.019	0.02
	α (mg g^−1^ min^−1^)	325.64	168.8	148.17

**Table 2:** Kinetic model constants and correlation coefficients for the adsorption of CR dye onto CCs at different temperatures.

Kinetic Models	Parameters	Temperatures			
		25 °C	35 °C	45 °C	55 °C
	q_e.exp_ (mg g^−1^)	333.11	433.33	462	494.44
pseudo- first-order	R^2^	0.957	0.973	0.978	0.958
	q_e.cal_ (mg g^−1^)	230.57	288.07	313.47	322.18
	K_1_ (10^−4^) (min^−1^)	6.909	6.909	6.909	11.151
	Δq_e_ (%)	10.88	12.67	12.15	13.17
pseudo- second-order	R^2^	0.99	0.993	0.992	0.991
	q_e,cal_ (mg g^−1^)	333.33	434.78	476.19	500
	K_2_ (10^−5^) (g·mg^−1^·min^−1^)	1.64	1.7	1.73	1.81
	Δq_e_ (%)	0.02	0.13	1.16	0.42
Elovich	R^2^	0.963	0.963	0.937	0.94
	β (g·mg^−1^)	0.027	0.021	0.02	0.02
	α (mg g^−1^ min^−1^)	36.24	64.72	96.36	148.17

**Table 3:** Kinetic model constants and correlation coefficients for the adsorption of CR dye onto CCs at different dye concentrations.

Kinetic Models	Parameters	Dye Concentrations			
		400 mg L^−1^	500 mg L^−1^	600 mg L^−1^	1000 mg L^−1^
q_e.exp_ (mg g^−1^)		398	474.67	555.33	760.44
pseudo- first-order	R^2^	0.972	0.975	0.989	0.879
	q_e.cal_ (mg g^−1^)	240.99	279.58	310.38	429.64
	K_1_ (10^−4^) (min^−1^)	13.82	9.21	9.21	6.91
	Δq_e_ (%)	13.95	14.53	16.67	15.38
pseudo- second-order	R^2^	0.998	0.996	0.997	0.99
	q_e,cal_ (mg g^−1^)	400	476.19	555.56	769.23
	K_2_ (10^−5^) (g·mg^−1^·min^−1^)	3.97	2.58	2.12	0.88
	Δq_e_ (%)	0.18	0.11	0.02	0.41
Elovich	R^2^	0.963	0.959	0.961	0.913
	β (g mg^−1^)	0.023	0.021	0.019	0.017
	α (mg g^−1^ min^−1^)	111.97	207.29	325.64	1874.6

**Table 4:** Parameters of intraparticle diffusion model using different pH for adsorption of CR dye onto CCs.

	Parameters	pH		
		4	7	9
Whole processes	R^2^	0.979	0.988	0.989
	k_i_	5.19	5.19	5.18
1st	K_i.1_	11.26	9.72	9.54
2nd	K_i.2_	5.5	5.24	5.17
3rd	K_i.3_	1.15	1.26	1.27

**Table 5:** Parameters of intraparticle diffusion model for adsorption of CR dye onto CCs at different temperatures.

	Parameters	Temperatures			
		25 °C	35 °C	45 °C	55 °C
Whole processes	R^2^	0.976	0.984	0.987	0.989
	k_i_	3.33	4.37	4.96	5.18
1st	K_i.1_	5.9	6.25	6.43	9.54
2nd	K_i.2_	2.84	4.1	5.06	5.17
3rd	K_i.3_	0.95	1.2	1.21	1.27

**Table 6:** Parameters of intraparticle diffusion model for adsorption onto CCs using different CR dye concentrations.

	Parameters	Dye Concentrations (mg L^−1^)			
		400	500	600	1000
whole processes	R^2^	0.988	0.986	0.979	0.989
	k_i_	4.7	4.88	5.19	5.55
1st	K_i.1_	6.59	7.9	11.26	11.99
2nd	K_i.2_	4.46	4.63	5.5	5.51
3rd	K_i.3_	1.91	1.41	1.15	0.96

**Table 7:** Adsorption isotherm constants of CR dye by CCs.

Isotherm Model	Parameters	
Langmuir	R^2^	0.98
	K_L_ (L mg^−1^)	0.008
	q_max_ (mg g^−1^)	666.67
	R_L_	0.217–0.111
Freundlich	R^2^	0.917
	K_f_ (L mg^−1^)	62.11
	1/n	0.35
Temkin	R^2^	0.92
	K_T_ (L mg^−1^)	0.08
	B (J mol^−1^)	149.74
D-R	R^2^	0.807
	X_max_ (mg g^−1^)	520.04
	β (mol^2^ J^−2^)	0.0009
	E (kJ mol^−1^)	0.023

**Table 8:** Parameters of thermodynamic of adsorbing CR dye by CCs.

Thermodynamic Parameters	Temperature (K)	
∆G° (KJ mol^−1^)	298	−0.55
	308	−2.45
	318	−3.19
	328	−4.21
ΔH° (kJ mol^−1^)	34.49	
ΔS° (J K^−1^ mol)	118.48	

**Table 9:** Comparison of adsorption capacity of CCs with other adsorbents for the removal of CR dye.

Adsorbent	Q_max_ (mg g^−1^)	Temperature °C	Dye Concentration mg L^−1^	Adsorbent Dose (g)	pH	Ref.
Tunics of the corm of the saffron	6.2	25	50–500	0.5	10	[46]
Quaternized chitosan/chitosan cationic polyelectrolyte microsphere	1500	25	0–1000	0.075	5	[47]
Activated carbon coffee waste	90.90	25	50 mg	0.1	3	[48]
xanthated chitosan/cellulose sponges	289.855	30	6.25–200	0.05	6	[49]
Chitosan and Laponite based nanocomposite	390.3	30	500	1	6	[50]
CCs	760.44	55	1000	0.05	4	Present study

## Data Availability

The data presented in this study are available on request from the corresponding author.

## References

[1] Shariatinia Z., Jalali A.M. (2018). Chitosan-based hydrogels: Preparation, properties and applications. Int. J. Biol. Macromol..

[2] Vakili M., Rafatullah M., Salamatinia B., Abdullah A.Z., Ibrahim M.H., Tan K.B., Gholami Z., Amouzgar P. (2014). Application of chitosan and its derivatives as adsorbents for dye removal from water and wastewater: A review. Carbohydr. Polym..

[3] Mohamed N.A., Al-Harby N.F., Almarshed M.S. (2021). Effective removal of Basic Red 12 dye by novel antimicrobial trimellitic anhydride isothiocyanate-cross linked chitosan hydrogels. Polym. Polym. Comp..

[4] Buthelezi S.P., Olaniran A.O., Pillay B. (2012). Textile dye removal from wastewater effluents using bioflocculants produced by indigenous bacterial isolates. Molecules.

[5] Crini G. (2006). Non-conventional low-cost adsorbents for dye removal: A review. Bioresour. Technol..

[6] Kadirvelu K., Kavipriya M., Karthika C., Radhika M., Vennilamani N., Pattabhi S. (2003). Utilization of various agricultural wastes for activated carbon preparation and application for the removal of dyes and metal ions from aqueous solutions. Bioresour. Technol..

[7] Annu S., Ahmed S., Ikram S., Ahmed S., Ikram S. (2017). Chitin and Chitosan: History, Composition and Properties, Chitosan.

[8] Ahmed S., Ikram S. (2015). Chitosan & its derivatives: A review in recent innovations. Int. J. Pharm. Sci. Res..

[9] Elmehbad N.Y., Mohamed N.A. (2021). Synthesis, characterization, and antimicrobial activity of novel N-acetyl, N’-chitosanacetohydrazide and its metal complexes. Int. J. Polym. Mater. Polym. Biomater..

[10] Sabaa M.W., Mohamed N.A., Mohamed R.R., Khalil N.M., Abd El Latif S.M. (2010). Synthesis, characterization and antimicrobial activity of poly (N-vinyl imidazole) grafted carboxymethyl chitosan. Carbohydr. Polym..

[11] Sabaa M.W., Mohamed N.A., Ali R., Abd El Latif S.M. (2010). Chemically induced graft copolymerization of acrylonitrile onto carboxymethyl chitosan and its modification to amidoxime derivative. Polym. Plast. Technol. Eng..

[12] Sabaa M.W., Mohamed N.A., Mohamed R.R., Abd El Latif S.M. (2011). Chemically induced graft copolymerization of 4-vinyl pyridine onto carboxymethyl chitosan. Polym. Bull..

[13] Mohamed N.A., Abd El-Ghany N.A. (2012). Synthesis, characterization, and antimicrobial activity of carboxymethyl chitosan-graft-poly(N-acryloyl,N’-cyanoacetohydrazide) copolymers. J. Carbohydr. Chem..

[14] Sabaa M.W., Abdallah H.M., Mohamed N.A., Mohamed R.R. (2015). Synthesis, characterization and application of biodegradable crosslinked carboxymethyl chitosan/poly(vinyl alcohol) clay nanocomposites. Mater. Sci. Eng. C.

[15] Abraham A., Soloman A., Rejin V.O. (2016). Preparation of chitosan-polyvinyl alcohol blends and studies on thermal and mechanical properties. Procedia Technol..

[16] Bahrami S.B., Kordestani S.S., Mirzadeh H., Mansoori P. (2003). Poly (vinyl alcohol)-chitosan blends: Preparation, mechanical and physical properties. Iran. Polym. J..

[17] Mohamed N.A., El-Ghany N.A.A., Abdel-Aziz M.M. (2021). Synthesis, characterization, anti-inflammatory and anti-Helicobacter pylori activities of novel benzophenone tetracarboxylimide benzoyl thiourea cross-linked chitosan hydrogels. Int. J. Biol. Macromol..

[18] Elmehbad N.Y., Mohamed N.A. (2021). Terephthalohydrazido cross-linked chitosan hydrogels: Synthesis, characterization and applications. Int. J. Polym. Mater. Polym. Biomater..

[19] Mohamed N.A., Abd El-Ghany N.A., Fahmy M.M. (2016). Novel antimicrobial superporous cross-linked chitosan/pyromellitimide benzoyl thiourea hydrogels. Int. J. Biol. Macromol..

[20] Elsayed N.H., Monier M., Youssef I. (2017). Fabrication of photo-active trans -3-(4-pyridyl)acrylic acid modified chitosan. Carbohydr. Polym..

[21] Mohamed N.A., Abd El-Ghany N.A. (2018). Novel aminohydrazide cross-linked chitosan filled with multi-walled carbon nanotubes as antimicrobial agents. Int. J. Biol. Macromol..

[22] Mohamed N.A., Abd El-Ghany N.A. (2019). Synthesis, characterization and antimicrobial activity of novel aminosalicylhydrazide cross linked chitosan modified with multi-walled carbon nanotubes. Cellulose.

[23] Mohamed N.A., Al-Harby N.F., Almarshed M.S. (2020). Enhancement of adsorption of Congo red dye onto novel antimicrobial trimellitic anhydride isothiocyanate-cross-linked chitosan hydrogels. Polym. Bull..

[24] Alharby N.F., Almutairi R.S., Mohamed N.A. (2021). Adsorption Behavior of Methylene Blue Dye by Novel Crosslinked O-CM-Chitosan Hydrogel in Aqueous Solution: Kinetics, Isotherm and Thermodynamics. Polymers.

[25] Surikumaran H., Mohamad S., Muhamad Sarih N. (2014). Molecular Imprinted Polymer of Methacrylic Functionalised β-Cyclodextrin for Selective Removal of 2,4-Dichlorophenol. Int. J. Mol. Sci..

[26] Mohamed N.A., Abd El-Ghany N.A. (2017). Synthesis, characterization, and antimicrobial activity of chitosan hydrazide derivative. Int. J. Polym. Mater. Polym. Biomater..

[27] Elmehbad N.Y., Mohamed N.A. (2020). Designing, preparation and evaluation of the antimicrobial activity of biomaterials based on chitosan modified with silver nanoparticles. Int. J. Biol. Macromol..

[28] Gonçalves J.O., Dotto G.L., Pinto L.A.A. (2015). Cyanoguanidine-crosslinked chitosan to adsorption of food dyes in the aqueous binary system. J. Mol. Liq..

[29] Burkhanova N., Yugai S., Pulatova K.P., Nikononvich G., Milusheva R.Y., Voropaeva N., Rashidova S.S. (2000). Structural investigations of chitin and its deacetylation products. Chem. Nat. Compd..

[30] Feng T., Zhang F., Wang J., Wang L. (2013). Application of Chitosan-Coated Quartz Sand for Congo Red Adsorption from Aqueous Solution. J. Appl. Polym. Sci..

[31] Salama H.E., Saad G.R., Sabaa M.W. (2016). Synthesis, characterization, and biological activity of cross-linked chitosan biguanidine loaded with silver nanoparticles. J. Biomater. Sci. Polym. Ed..

[32] El-Harby N.F., Ibrahim S.M.A., Mohamed N.A. (2017). Adsorption of Congo red dye onto antimicrobial terephthaloyl thiourea cross-linked chitosan hydrogels. Water Sci. Technol..

[33] Guarın J.R., Moreno-Pirajan J.C., Giraldo L. (2018). Kinetic Study of the Bioadsorption of Methylene Blue on the Surface of the Biomass Obtained from the Algae D. antarctica. J. Chem..

[34] Swan N.B., Abbas M., Zaini A. (2019). Adsrption of Malachite green and Congo red dyes from water: Recent progress and future outlook. Ecol. Chem. Eng..

[35] Banerjee S., Chattopadhyaya M.C. (2017). Adsorption characteristics for the removal of a toxic dye, tartrazine from aqueous solutions by a low cost agricultural by-product. Arab. J. Chem..

[36] Ojedokun A.T., Bello O.S. (2017). Kinetic modeling of liquid-phase adsorption of Congo red dye using guava leaf-based activated carbon. Appl. Water Sci..

[37] Md Ahsanul Haque A.N., Remadevi R., Wang X., Naebe M. (2020). Adsorption of anionic Acid Blue 25 on chitosan-modified cotton gin trash film. Cellulose.

[38] Kumar R., Ansari S.A., Barakat M.A., Aljaafari A., Cho M.H. (2018). A polyaniline@MoS2-based organic–inorganic nanohybrid for the removal of Congo red: Adsorption kinetic, thermodynamic and isotherm studies. New J. Chem..

[39] Lin C., Li S., Chen M., Jiang R. (2017). Removal of Congo red dye by gemini surfactant C12-4-C12 ∙ 2Br-modified chitosan hydrogel beads. J. Dispers. Sci. Technol..

[40] Titi Ojedokun A., Solomon Bell O. (2017). Liquid phase adsorption of Congo red dye on functionalized corn cobs. J. Dispers. Sci. Technol..

[41] Litefti K., Freire M.S., Stitou M., González-Álvarez J. (2019). Adsorption of an anionic dye (Congo red) from aqueous solutions by pine bark. Sci. Rep..

[42] Tahira I., Aslam Z., Abbas A., Monim-ul-Mehboob M., Ali S., Asghar A. (2019). Adsorptive removal of acidic dye onto grafted chitosan: A plausible grafting and adsorption mechanism. Int. J. Biol. Macromol..

[43] Zahir A., Aslam Z., Kamal M.S., Ahmad W., Abbas A., Shawabkeh R.A. (2017). Development of novel cross-linked chitosan for the removal of anionic Congo red dye. J. Mol. Liq..

[44] Bulut Y., Karaer H. (2015). Adsorption of Methylene Blue from Aqueous Solution by Crosslinked Chitosan/Bentonite Composite. J. Dispers. Sci. Technol..

[45] Jabar J.M., Odusote Y.A., Alabi K.A., Ahmed I.B. (2020). Kinetics and mechanisms of congo-red dye removal from aqueous solution using activated Moringa oleifera seed coat as adsorbent. Appl. Water Sci..

[46] Dbik A., Bentahar S., el Khomri M., el Messaoudi N., Lacherai A. (2020). Adsorption of Congo red dye from aqueous solutions using tunics of the corm of the saffron. Mater. Today.

[47] Cai L., Ying D., Liang X., Zhu M., Lin X., Xu Q., Cai Z., Xu X., Zhang L. (2021). A novel cationic polyelectrolyte microsphere for ultrafast and ultra-efficient removal of heavy metal ions and dyes. Chem. Eng. J..

[48] Lafi R., Montasser I., Hafiane A. (2019). Adsorption of Congo red dye from aqueous solutions by prepared activated carbon with oxygen-containing functional groups and its regeneration. Adsorpt. Sci. Technol..

[49] Xu X., Yu J., Liu C., Yang G., Shi L., Zhuang X. (2021). Xanthated chitosan/cellulose sponges for the efficient removal of anionic and cationic dyes. React. Funct. Polym..

[50] Xu G., Zhu Y., Wang X., Wang S., Cheng T., Ping R., Cao J., Lv K. (2019). Novel chitosan and Laponite based nanocomposite for fast removal of Cd(II), methylene blue and Congo red from aqueous solution. e-Polymers.

[51] Wang P., Yan T., Wang L. (2013). Removal of Congo Red from Aqueous Solution Using Magnetic Chitosan Composite Microparticles. BioResources.

